# Stochastic parcel tracking in an Euler–Lagrange compartment model for fast simulation of fermentation processes

**DOI:** 10.1002/bit.28094

**Published:** 2022-04-11

**Authors:** Cees Haringa, Wenjun Tang, Henk J. Noorman

**Affiliations:** ^1^ Biotechnology Department, Bioprocess Engineering Delft University of Technology Delft The Netherlands; ^2^ Department of Biotechnology, Bioprocess Engineering group, Faculty of Applied Sciences, Delft University of Technology Royal DSM Delft The Netherlands

**Keywords:** CFD, compartment model, Euler–Lagrange, fermentation, metabolic modeling

## Abstract

The compartment model (CM) is a well‐known approach for computationally affordable, spatially resolved hydrodynamic modeling of unit operations. Recent implementations use flow profiles based on Computational Fluid Dynamics (CFD) simulations, and several authors included microbial kinetics to simulate gradients in bioreactors. However, these studies relied on black‐box kinetics that do not account for intracellular changes and cell population dynamics in response to heterogeneous environments. In this paper, we report the implementation of a Lagrangian reaction model, where the microbial phase is tracked as a set of biomass‐parcels, each linked with an intracellular composition vector and a structured reaction model describing their intracellular response to extracellular variations. A stochastic parcel tracking approach is adopted, in contrast to the resolved trajectories used in CFD implementations. A penicillin production process is used as a case study. We show good performance of the model compared with full CFD simulations, both regarding the extracellular gradients and intracellular pool response, using the mixing time as a matching criterion and taking into account that the mixing time is sensitive to the number of compartments. The sensitivity of the model output towards some of the inputs is explored. The coarsest representative CM requires a few minutes to solve 80 h of flow time, compared with approximately 2 weeks for a full Euler–Lagrange CFD simulation of the same case. This alleviates one of the major bottlenecks for the application of such CFD simulations towards the analysis and optimization of industrial fermentation processes.

## Graphical abstract

Compartment models (CMs) provide a rapid method to simulate the impact of substrate heterogeneity on industrial bioprocesses. By incorporating structured metabolic models in a CM wherein the biomass population is represented by discrete parcels, predictions on the microbial response to substrate heterogeneity can be made which are close to full Computational Fluid Dynamics simulations, but with vastly reduced computational expenses. The workflow and performance are shown for a penicillin production process.

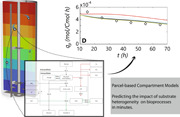

## 1 INTRODUCTION

Assessing the impact of environmental heterogeneity in industrial fermentations is a challenging aspect of process development. The disparity between timescales of nutrient uptake and of mixing may lead to nutrient gradients (Oosterhuis & Kossen, 1984) which may impact performance (Enfors et al., 2001; Larsson et al., 1996) and pose a scale‐up risk. From the cellular perspective, heterogeneous environments translate to temporal fluctuations, which experimental “scale‐down simulation” studies aim to replicate (Lara et al., 2006; Neubauer & Junne, 2010; Wang et al., 2015). Ideally, scale‐down studies represent the (expected) large‐scale environment, but quantifying this environment is not trivial for existing fermentors, let alone conceptual designs. Due to experimental limitations, quantification of the large‐scale environment often relies on simulations, combining Computational Fluid Dynamics (CFD) with “Computational Reaction Dynamics” (CRD). To incorporate the adaptation of microbes to fluctuating conditions, population balances (Morchain et al., 2013, 2016; Pigou & Morchain, 2014) or Lagrangian reaction models with biomass represented by virtual particles (parcels) are used (Lapin et al., 2004, 2006). While CFD considers detailed hydrodynamics, the computational burden constrains the use of combined CFD‐CRD. Even with considerable simplifications, weeks of simulation time may be required for a fed‐batch process.

Compartment models (CMs) form a middle ground between ideal reactor models and full CFD. Originally they were based on experimental data (Oosterhuis & Kossen, 1984; Vrábel et al., 1999, 2001), now CFD is typically used as a basis (Bezzo et al., 2003, 2004; Delafosse et al., 2014). Combined with black‐box kinetics (Nadal‐Rey et al., 2021, 2020; Spann, Gernaey, et al., 2019; Spann, Glibstrup, et al., 2019; Tajsoleiman et al., 2019), large‐scale gradients are estimated in seconds. However, black‐box kinetics assume instantaneous equilibrium between intra‐ and extracellular conditions, which is questionable. As for CFD, more realistic reaction dynamics can be incorporated via population balances (Pigou & Morchain, 2014; Pigou et al., 2017) or parcel‐based models. A methodology for parcel tracking in CM exists (Delafosse et al., 2015), but biokinetics were not included. In this study we implement reaction dynamics in a CM with parcel tracking. We focus on the technical implementation and benchmarking against CFD simulations. Whilst a considerable reduction in computation time is already evident, further numerical optimization is a subject for further study.

## 2 MODEL SETUP

We present a CFD‐based CM with the biomass phase represented by computational particles, called parcels (Haringa et al., 2017), each representing $C_{x,p}=C_{x}\cdot \rho_{l}V_{T}∕N_{p}\;g$ biomass, with $C_{x}$ the overall biomass concentration, $V_{T}$ the liquid‐filled reactor volume, and $N_{p}$ the number of parcels. Although CM simulations differ from CFD, we adhere to the terminology “Eulerian” and “Lagrangian” to distinguish fields (liquid phase) and parcels (biomass phase; Delafosse et al., 2015). Penicillin production in a $54\;m^{3}$ stirred fermentor is used as a case study; the CFD simulations underlying the CM were previously described by Haringa et al. (2016, 2018).

### 2.1 Compartment generation and flux calculation

**Figure 1:**
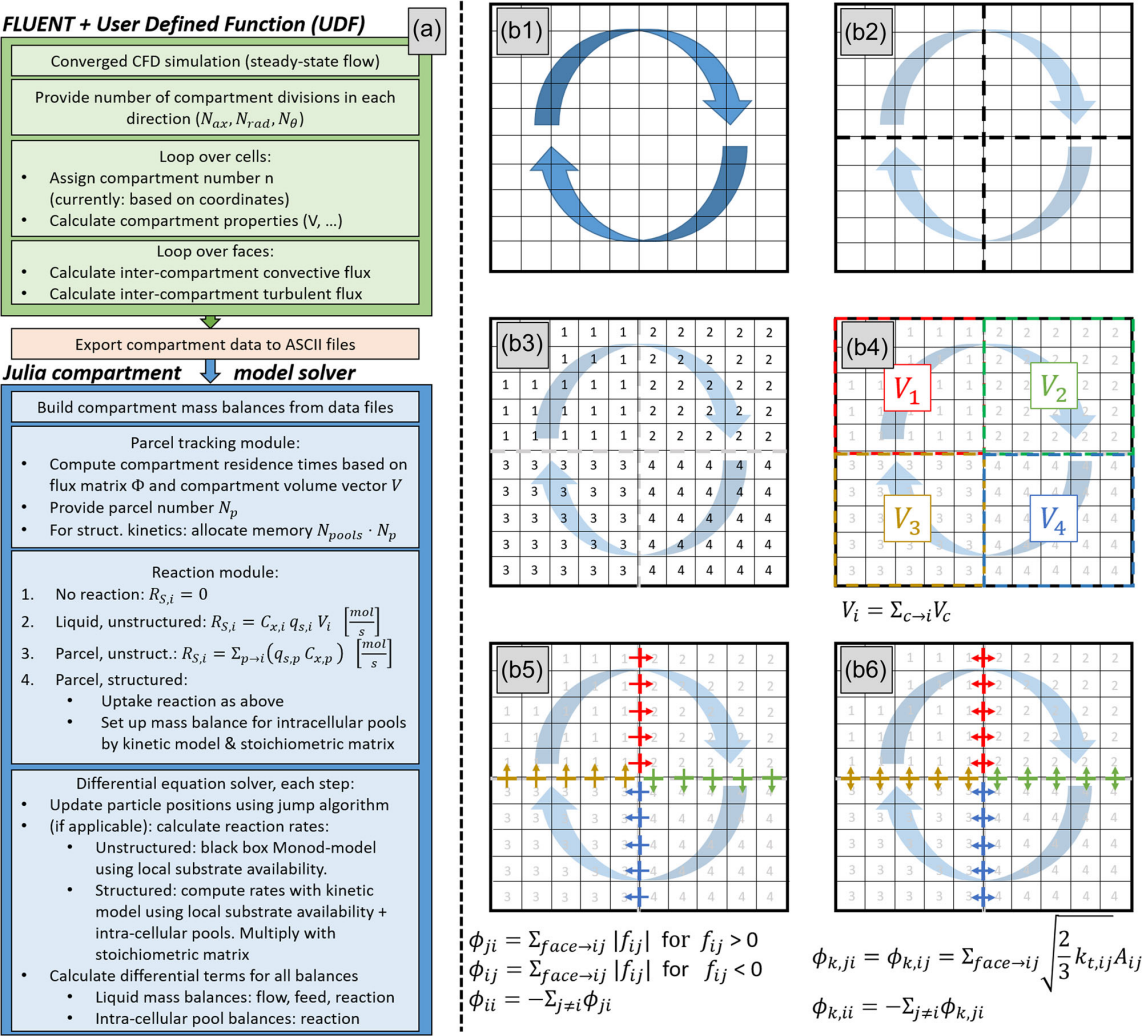
(a) Compartment modeling workflow. (Green boxes) Generation of compartments in ANSYS FLUENT. (Orange) Transfer from FLUENT to Julia. (Blue) Setup and solving of the compartment model in Julia, for different reaction coupling scenarios. (b) Illustration of compartment generation algorithm in ANSYS FLUENT. (b1) CFD Solution. (b2) Define (geometric) compartment division. (b3) Loop over cells to assign compartment number n. (b4) Loop over cells to compute compartment aggregate quantities, for example, compartment volume. (b5) Loop over faces to compute convective fluxes through compartment interfaces, as a sum of gridcell‐face fluxes on said interface. (b6) Loop over faces to compute turbulent fluxes through compartment interfaces, as the sum of gridcell‐face fluxes based on turbulent kinetic energy on said interface. CFD, Computational Fluid Dynamics

Compartments are generated by clustering gridcells in CFD solver ANSYS FLUENT through a user‐defined function (UDF). A homogeneous cylindrical compartment grid with $N_{ax}$, $N_{r}$, $N_{\theta }$ divisions in the respective dimensions is used (Delafosse et al., 2014); $N_{c}=N_{ax}\cdot N_{r}\cdot N_{\theta }$ is the total number of compartments. The cases in this study are codified by the number of divisions as $A\left[N_{ax}\right]R\left[N_{r}\right]T\left[N_{\theta }\right]$. The compartment generation procedure is summarized in Figure 1a (green box) and graphically outlined in Figure 1b. A compartment number is assigned to each gridcell (Figure 1b3) based on spatial coordinates. Compartment properties, for example, volume $V_{i}$, are summed for each compartment in a cell‐loop (Figure 1b4). The convective intercompartment liquid‐phase mass flux $\phi_{c,ij}$ (from compartments j to i) is computed by summing the fluxes $f_{c,\mathrm{face}}$ over all gridcell‐faces on the compartment interface where the flux direction is j to i; $\phi_{c,ji}$ from i to j is computed over the faces with reversed direction. Hence, $\phi_{c,ij}\neq \phi_{c,ji}$ (Figure 1b5). Together, the fluxes form the intercompartment convective flux matrix $\Phi_{c}$ (Equation 1) where the negative diagonal term $\phi_{ii}$ contains the flux out of compartment i, and the other nonzero entries in a row indicate fluxes into compartment i from neighbors (Delafosse et al., 2015).

(1) $$\begin{array}{c} \Phi_{c}=\left[\begin{array}{cccc} \phi_{c,11} & \phi_{c,12} & \text{…} & \phi_{c,1N_{c}}\\ \phi_{c,21} & \phi_{c,22} & \text{…} & \phi_{c,2N_{c}}\\ \text{⋮} & \text{⋮} & \text{⋱} & \phi_{c,3N_{c}}\\ \phi_{c,N_{c}1} & \phi_{N_{c}2} & \text{…} & \phi_{c,N_{c}N_{c}} \end{array}\right]. \end{array}$$

The turbulent intercompartment fluxes, collected in turbulent flux matrix $\Phi_{t}$, are determined by summing the turbulent gridcell‐face flux $f_{t,\mathrm{face}}$ (computed using Equation 2) over the compartment interfaces (Delafosse et al., 2014). Here, $k_{t}$ is the turbulent kinetic energy. In contrast to convection, turbulence is assumed to be bidirectional, hence $\phi_{t,ij}=\phi_{t,ji}$ (Figure 1b6).

(2) $$f_{t,\mathrm{face}}=A_{\mathrm{face}}\cdot \sqrt{2k_{t,\mathrm{face}}/3}.$$

This direct computation approach of intercompartment fluxes from the CFD face‐fluxes results in excellent closure of the compartment mass balances. The stochastic parcel tracking method presented next is also compatible to other means of compartment generation, such as compartment clustering based on, for example, the velocity field (Bezzo et al., 2003, 2004; Tajsoleiman et al., 2019), as well as with unstructured compartment layouts. Parcel transport between compartments depends solely on fluxes and volumes, not on the interface shape. We do note that there is a risk of overestimating fluxes with rugged compartment interfaces, an issue that is further laid out in Supporting Information Appendix C, but does not currently apply.

Matrices $\Phi_{c}$ and $\Phi_{t}$ and further compartment information (e.g., volumes) are transferred to Julia v1.5 (https://julialang.org/) as textfiles for CM–CRD calculations. The CM is a system of ODEs describing the species balances of intra‐ and extracellular components. The current implementation considers the liquid and parcel phase, resulting in $N_{\mathrm{tot}}=N_{c}\cdot N_{\mathrm{liq}}+N_{p}\cdot N_{\mathrm{pool}}$ differential equations, with $N_{\mathrm{liq}}$ the number of liquid‐phase species, and $N_{\mathrm{pool}}$ the number of intracellular pools in the model. A schematic overview of the CM implementation in Julia is provided in Figure 1a (blue box); the steps are outlined in the next sections.

Currently, we focus on glucose as a substrate ($N_{\mathrm{liq}}=1$). For penicillin production oxygen limitations may also play a role. As discussed in Haringa et al. (2016), no oxygen data were available for the studied process, and limitations were seemingly absent in a $150\;m^{3}$ fermentor. Considering this combined with the focus on Lagrangian implementation in CM, that the underlying CFD did not include oxygen, and that the biokinetic model is not configured for oxygen limitation, its impact is omitted. A more complete physical process description will be considered in future work.

### 2.2 Parcel position updating

Parcel positions are updated within the ordinary differential equation (ODE)‐update function every timestep. Compared with precomputed position vectors (Delafosse et al., 2015) there is a memory benefit and small performance penalty. Parcel transport consists of two steps: (1) determining whether a parcel p leaves compartment i, and (2) determining the destination compartment j in case it does. The first is determined by the compartment residence time: $\tau_{i}=V_{i}∕|\phi_{ii}|$, where $|\phi_{ii}|$ is the absolute total flowrate (convective plus turbulent) leaving compartment i, drawn from Φ. This links parcel transport to liquid flow, assuming that parcels are ideal flow‐followers (representing micron‐sized cells with low Stokes numbers). Assuming ideal mixing in each compartment, the probability of parcel p leaving compartment i in timespan Δt equals $P_{\mathrm{jump}}\left(p,i\right)=1-\text{exp}\left(-\Delta t∕\tau_{i}\right)$. For step (2), the relative probability for jumping to compartment j is $P_{\mathrm{dest},i}=\phi_{ji}∕|\phi_{ii}|$, with $\phi_{ji}$ the flowrate from i to j. Practically, the two steps can be combined by a “jump quantifier” $Q_{\mathrm{jump}}$ as shown in pseudocode‐algorithm 1; if $Q_{\mathrm{jump}}$ is negative, the parcel stays in place. If it is positive, $Q_{\mathrm{jump}}$ ranges from 0 to 1 and determines the destination compartment.

**Table:** 

**Algorithm 1. Pseudocode for the jump determination algorithm used in the CM model**
1: ψ=rand(1) #draw uniform random number between 0 and 1
2: $Q_{\mathrm{jump}}=\frac{P_{\mathrm{jump}}\left(p,i\right)-\psi }{P_{\mathrm{jump}}\left(p,i\right)}$ # jump quantifier 3: if $Q_{\mathrm{jump}}=<0$ not jumping
4: else $Q_{\mathrm{jump}}>0$ jump, determine destination
5: if $P_{\mathrm{dest},1}>=Q_{\mathrm{jump}}$ jump to 1st neighbor
6: elseif $\left(P_{\mathrm{dest},1}+P_{\mathrm{dest},2}\right)>=Q_{\mathrm{jump}}$ jump to second neighbor
7: elseif $\left(P_{\mathrm{dest},1}+P_{\mathrm{dest},2}+\text{⋯}+P_{\mathrm{dest},m}\right)>=Q_{\mathrm{jump}}$ jump to mth neighbor

As parcel transport is based on the same fluxes as liquid transport, mixing based on the parcel concentration should yield the same results as for a liquid tracer. We validated the implementation by confirming that the mixing time measured from both perspectives is equal (see Section 3.1).

### 2.3 Mass balances and reaction calculations

For liquid‐phase species, the mole balances are given by Equation (3), with $F_{s}$ the feed vector (0 in nonfeed compartments) and $R_{s}$ the reaction vector.

(3) $$dC_{s,c}/dt=\left(\Phi /V_{c}\right)\cdot C_{s,c}+F_{s}/V_{c}-R_{s,c}\;(\text{mol/kg/}s).$$

Depending on the model setup, $R_{s,c}$ is computed in different ways, with $R_{s,i}$ the reaction vector entry associated with compartment i:

- If only mixing is studied, $R_{s,i}=0$.
- If unstructured liquid‐phase kinetics are used, $R_{s,i}=C_{x}\cdot q_{s,i}$ with $q_{s,i}$ computed from the local substrate concentration.
- For parcel‐based kinetics, $R_{s,i}$ is computed as the sum of the uptake by all parcels p residing in gridcell i (Equation 4), with $q_{s,p}$ the uptake and $C_{x,p}$ the quantity of biomass related to parcel p.

For parcel‐based kinetics, the kinetic equations are calculated for each parcel. For an unstructured model, uptake rate $q_{s,p}$ is solely a function of $C_{s,i}$, with i the compartment containing p. For structured kinetics, both uptake rate $q_{s,p}$ and other (intracellular) rates $r_{p}$ may be a function of $C_{s,i}$ and intracellular metabolite‐ and enzyme pools $X_{p}$ linked to p. The balances of the intracellular metabolite pools associated p are determined by multiplication of reaction vector $r_{p}$ with stoichiometric matrix S (Equation 5), here, $\mu \cdot X_{p}$ represents pool dilution due to growth. For enzymatic pools, other equations may apply, according to the specific model formulation.

(4) $$R_{s,i}=\left(\Sigma_{p\;\to \;i}\left(q_{s,p}\cdot C_{x,p}\right)\right)/V_{c,i}\;\left(\text{mol}/\text{kg}/s\right),$$

(5) $$dX_{p}∕dt=S\cdot r_{p}-\mu \cdot X_{p}\;\left(\mu \text{mol}/g_{x}/h\right).$$

The resulting set of equations is solved with the dynamic stepsize Bogacki–Shampine 3/2 method (Bogacki & Shampine, 1989) implemented in *differentialequations.jl* (Rackauckas & Nie, 2017); this method balances accuracy and speed (Supporting Information Appendix B). The solver was controlled using the relative tolerance RelTol and maximum timestep size $\Delta t_{\max}$. We note the best solver may vary depending on the problem size and stiffness.

### 2.4 Geometry and setup

The CM is applied to a $54\;m^{3}$ penicillin production process. The stirred tank has diameter T=3 m and liquid‐filled height $H_{l}=7.7\;m$. In this model development stage, the broth is assumed to behave as a single phase, Newtonian liquid; inclusion of aeration and rheology add complexity, but do not affect the general modeling approach. The bottom impeller (8 blade Rushton) has diameter D=1.3 m and off‐bottom clearance C=1.3 m, the top impeller (6 blade Rushton) D=1.3 m and mutual impeller clearance ΔC=3 m. The stirring speed is 98 RPM. The frozen flow k−ϵ CFD model used for CFD is described in Haringa et al. (2016).

### 2.5 Modeling steps

The CM was used in four steps, described below. We use $N_{\mathrm{ax}}=26$, $N_{\mathrm{rad}}=6$, $N_{\theta }=1$ (A26R6T1) as the base‐case compartment layout. Unless otherwise mentioned, $N_{p}=1000$, an integrator *RelTol* of 0.001 is used, and the maximum integrator timestep size $\Delta t_{\max}=0.03$.

#### 2.5.1 Mixing study

Mixing is studied by injecting a tracer at y=7.4 m,r=0.75 m. In CFD, a volume with diameter d=0.4 m was patched with $Y_{s}=1\;\text{kg}∕\text{kg}$ at t=0, using a frozen flowfield, and setting turbulent Schmidt number $\sigma_{Sc}=0.2$ (Haringa et al., 2016). For liquid‐phase mixing in the CM, $C_{s}$ is set to 1mol∕kg at t=0 in the injection‐containing compartment, $N_{p}=0$, and stepsize $\Delta t_{\max}=0.01$. Mixing time $\tau_{95}$ is the time after which $0.95<C_{s,\mathrm{probe}}∕\bar{C_{s}}<1.05$, measured by a point‐probe at y=0.25 m, r=0.75 m off‐center, 180° compared with the injection plane. Parcel tracking is validated by comparing Eulerian and Lagrangian mixing. Lagrangian mixing is quantified by releasing $N_{p}$ parcels in the injection compartment and monitoring the local parcel concentration $C_{p,i}=N_{p,i}∕V_{i}$. Instead of a probe, the coefficient of mixing (volume‐weighted coefficient of variation, Equation 6) is used to monitor volumetric mixing with $\tau_{95}$ registered when CoM<0.0283 (Hartmann et al., 2006).

(6) $$CoM\left(t\right)=\sqrt{\left(\frac{\Sigma_{i}{\left(\frac{C_{s,i}-\bar{C}}{\bar{C_{s}}}\right)}^{2}\Delta V_{i}}{\Sigma_{i}\Delta V_{i}}\right)}.$$

#### 2.5.2 Black‐box kinetics

Black‐box kinetics are used in two ways: coupled to the liquid phase, for comparison with CFD (Haringa et al., 2016), and coupled to the parcel phase, to validate the parcel‐based reaction implementation. The black‐box model considers only substrate uptake using Monod kinetics (Equation 7), with maximum uptake rate $q_{s,\max}=1.6\;m\text{mol/}g_{\mathrm{dw}}/h$ and affinity $K_{s}=7.8\;\mu \text{mol/kg}$ (de Jonge et al., 2011). The biomass concentration was fixed at $C_{x}=55\;g_{\mathrm{dw}}∕\text{kg}$. In the Lagrangian implementation, the biomass per parcel equals $C_{x,p}=C_{x}\cdot \left(V_{T}\cdot \rho_{l}\right)∕N_{p}$, with $V_{T}$ the total reactor volume. A constant glucose feed of $F=1.23\;g/m^{3}/s$ is set at y=7.4 m,r=0.75 m.

(7) $$q_{s}=q_{s,\max}\cdot \frac{C_{s}}{K_{s}+C_{s}}.$$

#### 2.5.3 Structured kinetics—chemostat

Structured kinetics are implemented using the 9‐pool penicillin model (Tang et al., 2017), described in Supporting Information Appendix D. The ATP mass balance is replaced by an algebraic formulation to improve stability (Haringa et al., 2018); this modification means $N_{\mathrm{pool}}=8$ (4 metabolic, 4 enzymatic). Chemostat‐mode is implemented by fixing the pool size of $X_{E,11}$, fixing $C_{x}=55\;g∕\text{kg}$, and setting a constant feed $F=1.23\;g∕m^{3}∕s$. Two parameter sets for uptake kinetics from Haringa et al. (2018) are used: “TU‐A,” with $k_{11}\cdot X_{E,11}=1.13\;m\text{mol/}g_{\mathrm{dw}}/h$ and $K_{s}=9.8\;\mu \text{mol/kg}$ (the 9‐pool model parameters of Tang et al.), and “TU‐B” with $k_{11}\cdot X_{E,11}=1.6\;m\text{mol/}g_{\mathrm{dw}}/h$ and $K_{s}=7.8\;\mu \text{mol/kg}$, the black‐box model parameters of de Jonge et al. (2011). A sensitivity study (based on “TU‐B”), reported in Supporting Information Appendix B for brevity, is conducted towards the penicillin production rate ($q_{p}$) and computation time, addressing the impact of $N_{c}$, $N_{p}$, $\Delta t_{\max}$, integrator tolerance and integration algorithm.

#### 2.5.4 Structured kinetics—fed‐batch

In the fed‐batch setup, $X_{E,11}$ and $C_{x}$ do vary, but the total reactor volume $V_{T}$ remains fixed, due to the limitations of the underlying CFD. A feed profile (Figure 7a) is imposed, with the feed‐rate F, corrected for the fixed volume, being sampled every 30 s. A 60 h of process‐time is modeled, starting at t=10 h, with $C_{x,t=10}=14\;g/\text{kg}$ and using $K_{s}=9.8\;\mu \text{mol/kg}$.

## 3 RESULTS AND DISCUSSION

### 3.1 I: Flow and mixing

**Figure 2:**
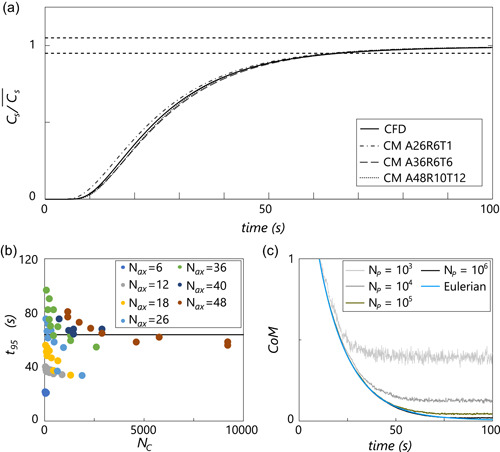
Mixing behavior in the compartment model. (a) Mixing curve (single‐point measurement) for several realizations of the compartment model compared with the CFD result. Note that the cases were selected based on agreement with the CFD simulation; more cases are discussed in Supporting Information Appendix A. (b) Point‐based mixing time versus number of compartments (colored by $N_{\mathrm{ax}}$) for the full range of tested compartment realizations. (c) Comparison between Eulerian (tracer‐based) and Lagrangian (parcel‐based) mixing time, using the coefficient of mixing, for various $N_{p}$. $N_{c}=156$ in these cases (A26R6T1). CFD, Computational Fluid Dynamics; CM, compartment model

Liquid‐phase mixing in the Eulerian CM is verified versus CFD results (Haringa et al., 2016). Mixing curves collected at the probe location (see Section 2.5.1) for selected cases are shown in Figure 2a; the 95% mixing time versus number of compartments $N_{c}$ is visualized in Figure 2b. In line with Delafosse et al. (2014), $\tau_{95}$ matches CFD well for large $N_{c}$, but ideally we want $N_{c}$ to be small to minimize computational demand. In the low $N_{c}$‐range, a broad scatter in $\tau_{95}$ is observed: few radial compartments $N_{r}$ result in an overestimation of $\tau_{95}$ (by underresolving the circulation), while few axial compartments $N_{\mathrm{ax}}$ leads to underestimation of $\tau_{95}$ (by underresolving axial mixing resistance). Properly balancing $N_{r}$ and $N_{\mathrm{ax}}$ can lead to a good solution in terms of $\tau_{95}$. Keep in mind that this solution is not “grid‐independent,” it is acquired by balancing out errors. Still, if the predicted magnitude of the gradient and subsequent metabolic calculations are not impacted by the low $N_{c}$, this forms a pragmatic, computationally manageable approach to model heterogeneity similar to the coarse manual compartmentalization used by Spann, Gernaey et al. (2019). We further explore the impact of $N_{c}$ in Section 3.3. Overall we conclude the mixing features observed in the CFD are well represented in the CM model, provided $N_{c}$ is chosen appropriately (or set large enough). A more elaborate mixing verification, including comparison to lab‐scale data (Jahoda et al., 2007), is presented in Supporting Information Appendix A.

The parcel tracking algorithm is validated by computing the parcel mixing time with $N_{p}=10^{3}-10^{6}$ in the base‐case CM. Figure 2c compares the tracer‐based and parcel‐based CoM‐curves, revealing excellent agreement for large $N_{p}$. For low $N_{p}$, statistical fluctuations in the local parcel concentration prohibit the 95% mixing threshold from being reached, but this is not necessarily problematic: if fluctuations are fast compared with the reaction timescale ($\tau_{\mathrm{rxn}}$), they do not propagate (significantly) in the reaction model. Importantly, the first 10 s of Figure 2c shows the rate of mixing is not affected by low $N_{p}$; deviations from the Eulerian curve only occur when stochastic effects set in. In Supporting Information Appendix A we show that the mixing rate deviates for large $\Delta t_{\max}$ due to a bias towards long compartment residence times. However, when kinetics are included, adaptive timestepping keeps Δt small, sufficiently mitigating this effect (Supporting Information Appendix B).

### 3.2 II: Black‐box kinetics

**Figure 3:**
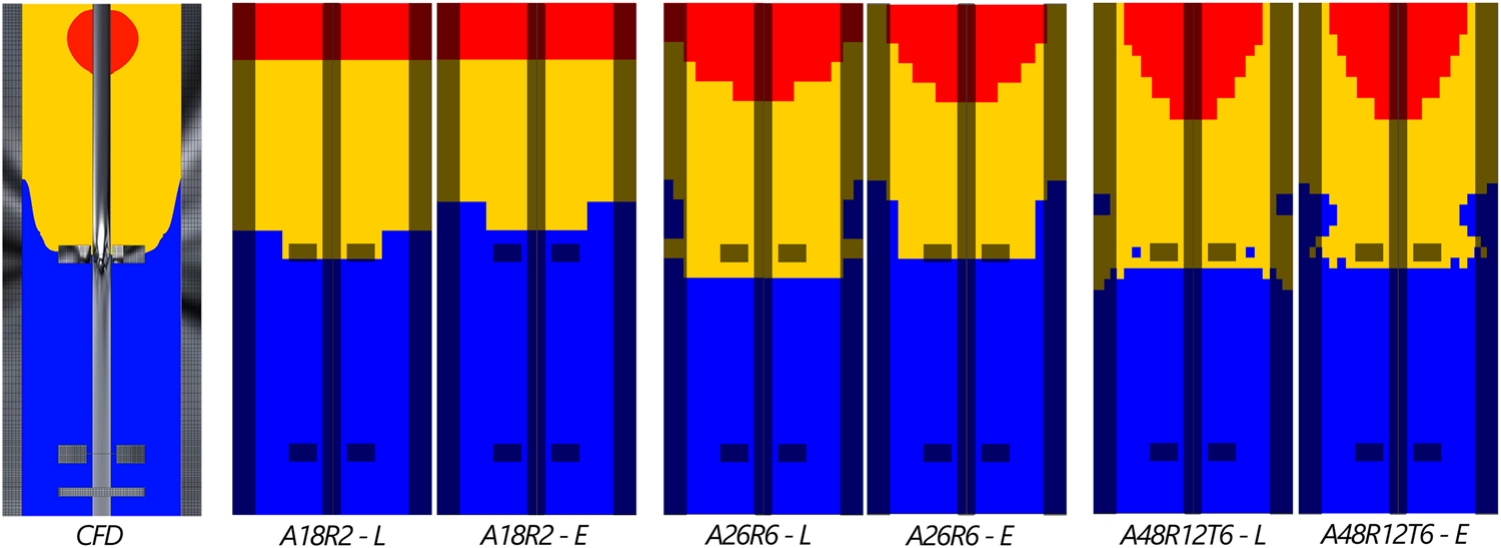
Regime analysis in the compartment model using Monod kinetics. (Left) Full CFD model ($\tau_{95}=63.8$). Compartment models layouts from left to right: A18R2T1 ($N_{p}=1000$, $\tau_{95}=56.2$), A26R6T1 ($N_{p}=1000$, $\tau_{95}=61.6$), A48R12T6 ($N_{p}=5000$, $\tau_{95}=68.2$)). “*L*” indicates the time‐averaged Lagrangian result, “*E*” the Eulerian result. (Red) Excess, $q_{s}∕q_{s,\max}>0.95$. (Blue) Starvation, $q_{s}∕q_{s,\max}<0.05$. (Yellow) Limitation, in between. CFD, Computational Fluid Dynamics

Black‐box Monod kinetics are implemented in both the Eulerian and Lagrangian frameworks, to verify the substrate gradient with respect to CFD. The (*E*)xcess/(*L*)imitation/(*S*)tarvation regime definition (Haringa et al., 2016) is used for visualization. Figure 3 compares the regime distribution for various compartment layouts, for both Eulerian and Lagrangian couplings, with CFD. The finite $N_{p}$ causes fluctuations in local biomass concentration for Lagrangian coupling, inducing spurious substrate concentration fluctuations and hence regime fluctuations. These fluctuations are averaged out over 1800 s (ca. 30 mixing times). With both coupling methods the regime distribution is reproduced to a satisfactory degree. The size of the *E* and *L* regime is slightly larger in the Lagrangian CM (quantified in Table 1 over a full 80 h process simulation), while the average residence time is somewhat lower (Table 2). These deviations are small, but may have some impact on the $q_{p}$‐results with a coupled metabolic model.

**Table 1:** Comparison of mean extracellular substrate concentration and the regime division for different $N_{p}$; other settings equal that of the base‐case

	$N_{p}=100$	$N_{p}=1000$	$N_{p}=5000$	CM (Euler)	CFD (Euler)
$\bar{C_{s}}$	4.23 ⋅ 10^−5^	4.17 ⋅ 10^−5^	4.15 ⋅ 10^−5^	3.92 ⋅ 10^−5^	3.44 ⋅ 10^−5^
$\bar{C_{s,p}}$	3.84 ⋅ 10^−5^	3.97 ⋅ 10^−5^	3.99 ⋅ 10^−5^	3.81 ⋅ 10^−5^	3.29 ⋅ 10^−5^
Excess (%)	9.0 ± 5.5	9.1 ± 2.0	8.9 ± 1.0	8.7	6.8
Limit. (%)	37.5 ± 10.6	35.7 ± 4.3	35.7 ± 2.4	33.9	36.2
Starv. (%)	53.5 ± 11.1	55.2 ± 4.6	55.4 ± 2.5	57.4	57.0

*Note*: The Lagrangian results are averaged over 80 h, the margin indicates 2 standard deviations. Cs  is the mean volumetric substrate concentration (mol/kg), Cs,p  the mean substrate concentration registered by parcels (mol/kg). For the Lagrangian cases, the regime distribution is reported from the parcel perspective. In the Eulerian case, $N_{p}=100$  parcels were tracked as passive concentration readers to compute $\bar{C_{s,p}}$.

Abbreviations: CFD, Computational Fluid Dynamics; CM, compartment model.

**Table 2:** Average regime residence times in CM and CFD simulations, registered per transition pattern

Case	$\bar{\tau_{\mathrm{reg}}}\;s$, CM (Lagrange)	$\bar{\tau_{\mathrm{reg}}}\;s$, CM (Euler)	$\bar{\tau_{\mathrm{reg}}}\;s$, CFD
LEL	3.09	3.01	3.65
LSL	5.06	5.65	9.37
ELE	3.13	3.54	4.67
ELS	5.36	5.67	6.45
SLE	5.61	5.92	5.39
SLS	2.06	2.07	3.77

*Note*: CM simulations were conducted with $N_{p}=1000$, 7200 s  flow‐time, and $\Delta t_{\max}=0.03\;s$.

Abbreviations: CFD, Computational Fluid Dynamics; CM, compartment model.

**Figure 4:**
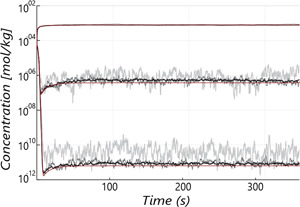
Oscillations in substrate concentration, measured at three different axial locations: at the feed point (top lines), at r=0.75 m,y=3.85 m (middle lines) and at r=0.75 m,y=0.25 m (bottom lines). (Light gray lines) $N_{p}=100$, (dark gray) $N_{p}=1000$, and (black) $N_{p}=\text{10,000}$. The red lines represent the Eulerian solution

The spurious oscillations in local substrate concentrations due to the finite $N_{p}$ are visualized in Figure 4 in three compartments; lower $N_{p}$ results in stronger oscillations. A consistent, positive offset in $C_{s}$ can be observed, which is more pronounced at low $N_{p}$. In brief (further discussed in Supporting Information Appendix B.2), this offset occurs because at low $N_{p}$, uptake only occurs in a subset of compartments (those containing parcels), raising $C_{s}$ in “empty” compartments, and consequently the average volumetric substrate concentration $\bar{C_{s}}$ (Haringa et al., 2017). Whether these spurious oscillations propagate into the intracellular response depends on the timescales; if the timescale of artificial extracellular oscillations matches the timescales related to intracellular pools, artificial intracellular oscillations may be observed, which could result in erroneous results (e.g., if there are irreversible or hysteresis effects in the metabolic model). Currently, some oscillations are observed in pool $X_{\mathrm{gly}}$ (especially with low $N_{p}$), but these do not substantially propagate to other intracellular pools due to their longer turnover times. A second effect observed for low $N_{p}$ is a discrepancy between the volumetric mean $\bar{C_{s}}$ and mean concentration registered by parcels, $\bar{C_{s,p}}$, because parcels by definition reside in compartments where uptake *is* active. The discrepancy strongly reduces for large $N_{p}$, but does not vanish completely for $\Delta t_{\max}=0.03$ as some inhomogeneity in the parcel distribution exists, by the long‐residence time bias discussed in Section 3.1. These observations are discussed in Supporting Information Appendix B.2.

**Figure 5:**
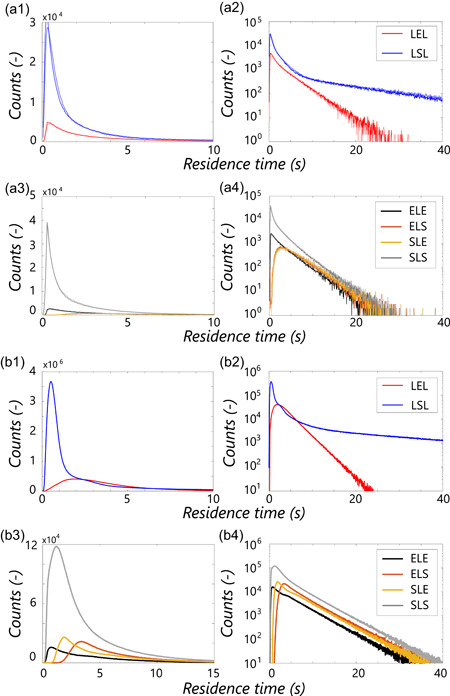
Regime analysis (Haringa et al., 2016) for the base‐case CM model (a), compared with CFD results (b). Results represent the nonnormalized number of counts. (a1, a2) CM results for the excess (red) and starvation regime (blue) with a linear and logarithmic *y*‐axis, respectively; dark lines represent Lagrangian reaction coupling, light lines Eulerian coupling. (a3, a4) Results for the limitation regime, discriminated by trajectory. (b) CFD results (Haringa et al., 2016); the difference in the number of counts (*y*‐axis) compared with (a) originates from differences in $N_{p}$ and timespan. CFD, Computational Fluid Dynamics; CM, compartment model

**Figure 6:**
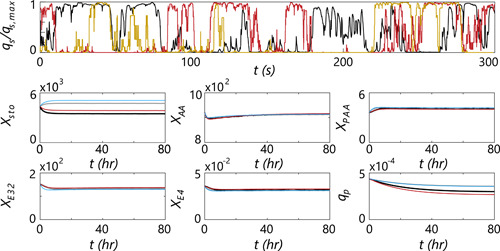
(Top) Example lifelines of specific glucose uptake rate (scaled with $q_{s,\max}$) for a parcel in the base‐case (red) and coarse model (A18R2, yellow) compared with the CFD case (black), using the chemostat setup. (Bottom) Pool dynamics of the CFD simulations versus CM base‐case. (Black) CFD, parameter set “TU‐B.” (Gray) CFD, parameter set “TU‐A.” (Red) CM, parameter set “TU‐B.” (Blue) CM, parameter set “TU‐A.” CFD, Computational Fluid Dynamics; CM, compartment model

For further comparison, lifelines are collected over 7200 s flowtime with sampling interval $\Delta t_{\mathrm{sample}}=0.06\;s$ in both the Eulerian and Lagrangian models (in the Eulerian model, parcels are passive tracers). Example lifelines are shown in Figure 6, top. The lifelines are subjected to the regime‐analysis method (Haringa et al., 2016) to compare flow phenomena between the CM and CFD, Figure 5. As in CFD, a moving average filter with a window of 0.36 s and a “fuzzy boundary” filter of ${\left(q_{s}∕q_{\max}\right)}_{E}=0.95\pm 0.01$ for excess and ${\left(q_{s}∕q_{\max}\right)}_{S}=0.05\pm 0.01$ are applied to remove short, low‐amplitude regime transitions (see Haringa et al., 2016 for details). The residence time distributions for the Lagrangian (dark lines) and Eulerian (light lines) nearly perfectly overlap (Figure 5a). Compared with CFD (Figure 5b), very similar features are observed, albeit with more prominent short‐time peaks (especially for *SLS* and *LSL*). Because of the ideal mixing assumption, parcels can instantly jump back and forth in CM, whereas in CFD a circulation trajectory through a regime has to be followed. These effects are also reflected in the mean residence times $\bar{\tau_{\mathrm{reg}}}$ (Table 2), which are similar, but except *SLE*, slightly shorter in the CM. Generally, $\bar{\tau_{\mathrm{reg}}}$ is slightly shorter in the Lagrangian implementation. This may reflect that, by taking up substrate, parcels introduce additional dynamics in their direct environment that somewhat affect the distributions.

### 3.3 III: Structured kinetics: Chemostat

With structured kinetics, 80 h of flowtime is simulated to establish steady‐state intracellular pools. The CM simulations are compared with CFD in Figure 6, bottom, with $N_{p}=1000$. Data were stored every 3600 s to minimize time spent on data writing, and averaged over all parcels. The base‐case CM with parameter set “TU‐A” shows a near‐perfect match with the CFD result, with a relative offset in $q_{p}$ of ca. 1%. This is despite an overestimation of the excess zone and underestimation of the starvation zone, indicating these impacts cancel each other out (details in Supporting Information Appendix B.6). The results with parameter set “TU‐B” show ca. 10% relative offset in the predicted $q_{p}$, indicating that for this case, the impact of the larger excess zone/higher $\bar{C_{s}}$ observed by parcels in the CM dominates (Table 2, further discussed in Supporting Information Appendix B.2). These results indicate that selection of the compartment layout only using $\tau_{95}$ may lead to a margin of error in predictions; a more thorough evaluation, aimed at matching the (black‐box) regime distribution, may provide more accurate results. This will be investigated in future work. In Table 3, results are shown for several other compartment layouts with similar $\tau_{95}$, revealing the limited impact of $N_{c}$ and $N_{p}$. While CFD required around 1 day of computation *per hour* of flow time (with frozen flow, Δt=0.03 and $N_{p}=2500$), the CM requires slightly more than an hour to run *80 h of flow time* for the base‐case, and ca. 3 min for the coarsest implementation. Naturally, the numbers will be dependent on the model complexity: while low $N_{p}$ facilitates a fast runtime, it is not suitable to study the potential emergence of population heterogeneity; this requires larger $N_{p}$ with due computational cost. A wide range of model settings are analyzed in Supporting Information Appendix B.

**Table 3:** Performance of various compartment realizations in predicting $q_{p}$ in chemostat configuration

Compartments	Parcels	Runtime (s)	$q_{p}\left(\mathrm{end}\right)$	%diff.CFD
CFD(TU‐A)	2500	$>10^{6}$	$3.57\cdot 10^{-4}$	–
A26R6T1	1000	4363	$3.54\cdot 10^{-4}$	−0.8
A18R2T1	36	183	$3.54\cdot 10^{-4}$	−0.9
CFD(TU‐B)	2500	$>10^{6}$	$2.99\cdot 10^{-4}$	–
A26R6T1	1000	4247	$2.67\cdot 10^{-4}$	−10.5
A48R10T6	1000	29,602	$2.78\cdot 10^{-4}$	−6.9
A18R2T1	1000	3973	$2.74\cdot 10^{-4}$	−8.3
A18R2T1	36	201	$2.79\cdot 10^{-4}$	−6.6

*Note*: In all cases, the total flowtime is 80 h, $\Delta t_{\max}=0.03\;s$, *RelTol* = 0.001. The top rows match the Computational Fluid Dynamics (CFD) simulation “TU‐A” from (Haringa et al., 2018), the bottom rows match CFD simulation “TU‐B.” More cases are reported in Supporting Information Appendix B.

### 3.4 IV: Structured kinetics: Fed‐batch

**Figure 7:**
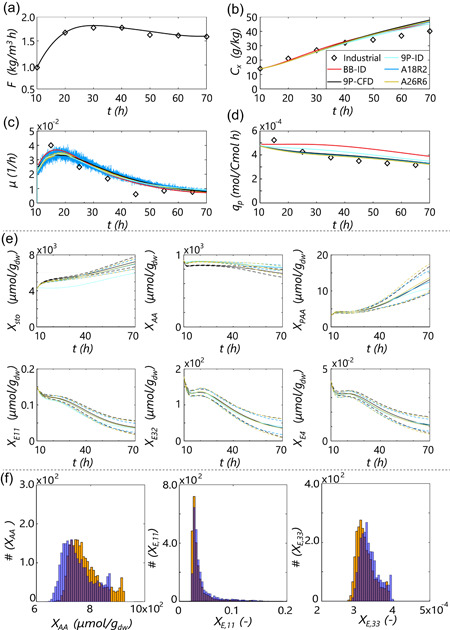
Dynamics of a fed‐batch simulation for two compartment realizations with stochastic parcel tracking and parcel‐based kinetics (base‐case and A18R2 with $N_{p}=36$), compared with the full CFD simulation, an ideal‐mixed model, and plant data (Haringa et al., 2018). (a) Imposed feed profile. (b) Biomass concentration (legend applies to c and d, too). (c) Growth rate. (d) Specific penicillin production rate. (e) Parcel‐averaged intracellular pools. The dashed lines indicate ±1 standard deviation. (f) Histograms of three pools (amino acids, transporter enzymes, and penicillin production enzymes) showing population heterogeneity at the end of the process. (Orange) Current simulation. (Transparent blue) CFD simulation from prior work (Haringa et al., 2018). CFD, Computational Fluid Dynamics

To conclude, we compare the CM with fed‐batch CFD (Haringa et al., 2018). We are currently subject to the same simplifications as in CFD, particularly assuming a constant volume. In future work, this limitation may be lifted, for example, by stepwise updating of the compartment volumes (Nadal‐Rey et al., 2021). Biomass per parcel $C_{x,p}$ and the glucose transporter concentration $X_{11}$ are dynamic pools in the fed‐batch simulation. Figure 7 compares CFD, the CM base‐case in fed‐batch mode, and a low‐resolution CM‐case (A18R2T1, $N_{p}=36$, $\Delta t_{\max}=0.03\;s$). In addition, ideally mixed black‐box and 9‐pool model simulations are added. The industrial data shows that in the first 20h, $q_{p}$ rapidly drops (Figure 7c), despite the growth rate μ=0.03 being near the optimum (Douma et al., 2011, 2010) (Figure 7c). This drop is attributed to the heterogeneous substrate concentration in the tank. By definition, this is not accounted for in the ideally mixed simulations (BB‐ID and 9P‐ID), which predict a steady $q_{p}$ until μ drops due to increasing $C_{x}$ at a constant feed rate F (Figure 7a). Overall, 9P‐ID predicts a lower $q_{p}$ than BB‐ID because the parameterization of the structured model gives a slightly lower maximum $q_{p}$ (Tang et al., 2017). The CM and CFD simulations do predict the initial drop in $q_{p}$, where the parcel‐bound kinetics, that allows parcels to be out of equilibrium with their surroundings, is essential to predict the rate of change in $q_{p}$ (instant adaptation of $q_{p}$ to local $C_{s}$ would induce a much larger $q_{p}$ drop; Haringa et al., 2016). Both the CM and CFD do overpredict $C_{x}$ slightly more than the ideally mixed models (Figure 7b). Because this is not the case for 9P‐ID compared with BB‐ID, it is likely a result of the CFD assumptions (e.g., constant volume, hence $\tau_{\mathrm{mix}}$) rather than model parameterization. This may be tested in future work if the dynamic volume is implemented. Important for the current scope is that the CM models, even with low resolution, are in excellent agreement with CFD for the model under consideration. Due to the stochasticity induced by low $N_{p}$, the growth rate (Figure 7c) is more strongly oscillating in the CM simulations, but no systemic offset is observed.

If we consider the intracellular pools (Figure 7e), we see good agreement between CM and CFD, with minor offsets in mean pool size and the standard deviation, the latter being representative of population heterogeneity. The emergence of population heterogeneity in the base‐case CM is visualized in figure 7f, showing the distribution of the amino acid pool, glucose transporter pool, and penicillin‐producing enzyme pool. As in Figure 7e, we observe good qualitative agreement with a minor quantitative offset compared with CFD. As for the CFD approach, the CM approach is capable of making predictions regarding the emergence of population heterogeneity, although we must stress (as in Haringa et al., 2018) that the predictions regarding population heterogeneity have not been experimentally verified. At this point, they mostly serve to present hypotheses for experimental follow‐up, which we regard as an interesting future avenue.

## 4 CONCLUSIONS

We report a CM with stochastic parcel tracking to represent the biotic phase to allow coupling of structured multipool models, akin to Lagrangian tracking in CFD simulations. The extracellular conditions experienced by microbes in these models can be monitored and used in scale‐down design. The use of CM provides a strong computational gain; even simplified Euler–Lagrange CFD can take weeks for a full batch—compared with minutes for the coarsest CM. While the substrate gradient layout matches well, the simplified hydrodynamics in CM lead to slight differences in the substrate distribution compared with CFD, resulting a relative offset in the microbial response of up to 10% depending on the kinetic parameter set. The compartment layouts were selected based on a single‐point mixing criterion, which may not fully reflect mixing and hence substrate distribution in the whole reactor. We expect that compartment selection based on matching the substrate distribution (with black‐box kinetics) reduces the offset. The CM simulations run faster than real‐time, opening up new applications in, for example, process optimization, that are out of reach for full CFD. A number of physical simplifications were made in the model setup, equaling those in the CFD model. Future extensions may aim at improving the physical process model, by including, for example, the gas phase, mass transfer, and volume changes in fed‐batch processes (Nadal‐Rey et al., 2021). Further gains in runtime may be possible by numerical optimization, considering solvers that are better suited for the stochastic nature of parcel tracking than regular ODE‐solvers, or making use of the notion that successive exponential decay processes aggregate to a Poisson process. The current implementation focused on a simple, geometrical compartment division. This can be improved using phenomenological compartmentalization, for example, based on the flowfield (Tajsoleiman et al., 2019) or substrate distribution, or using relevant timescales in the process to guide compartmentalization. The stochastic parcel tracking implementation is compatible with other means of compartmentalization, as it is based only on compartment volumes and intercompartment fluxes.

## Supporting information

Supplementary Information
