# Supplementary material for: Stochastic parcel tracking in an Euler–Lagrange compartment model for fast simulation of fermentation processes
Source: Biotechnol Bioeng. 2022 Apr 11;119(7):1849–60. doi: 10.1002/bit.28094 (PMC9321588; doi:10.1002/bit.28094)
Supplement: Supplementary file 1 — Supplementary Information [file BIT-119-1849-s001.pdf]

Appendix A. Mixing time verification

The hydrodynamic performance of the compartment model was verified by comparing the mixing behavior observed in three stirred reactor configurations with full CFD simulations of the same systems. The following three test cases were studied:

- single Rushton impeller, full domain.
- dual Rushton impeller, 180° domain
- dual Rushton impeller (penicillin production), 180° domain

Note that all cases studied here have the simplification of single phase flow. We expect the methods to be equally applicable to multiphase flow. The flow patterns in the CFD simulation would be different in that case, but they can equally be translated to the appropriate inter-compartment flow maps.

Appendix A.1. Single impeller geometry

The single Rushton case is based on the geometry of Jahoda et al (Jahoda et al., 2007): $T = 0.29$ m, $H = T$, $C = T/3$ and $D = T/3$. The flow is solved in steady state with the multiple-reference frame (MRF) method ($N_{str} = 300$ RPM), *realizable* $k - \epsilon$ turbulence model, and 2nd order discretization. Since we are interested in the performance of the CM compared to CFD, no mesh dependency study was done. After flow convergence, the flow is frozen, and species tracking equations enabled. The simulation is switched to transient mode ($\Delta t = 0.01$ s). The tracer concentration development is monitored at a single probe point ($r = 9.5T/20, Y = T/4, \theta = 45^o$), the tracer pulse is injected at $t = 0$ at $r = T/4, Y = 0.28$ m$, \theta = 225^o$, both in line with the experimental setup. With this, the CFD mixing curve, reported in fig. B1, **C** is acquired.

The CFD domain is translated into a range of compartment layouts, with the following divisions: $N_{ax} = 3 - 48$, $N_r = 2 - 12$, $N_\theta = 6 - 18$. CM-mixing simulations using the same injection and detection locations are conducted in Julia. The observed τ_{95} for all compartment layouts is shown in fig. B1, **A**, with the dots colored by N_{ax}, and the black line representing the CFD benchmark. It is visible that, while slightly underestimating τ_{95}, the compartment mixing time becomes 'grid independent' for $N_C > 2500$, within the space of chosen compartment layouts. For low N_{ax}, the mixing time is strongly under-estimated because axial transport is not properly resolved, while a high N_{ax} with low N_r leads to a strong over-estimation of the mixing time, because of poor resolution of the circulation loop. N_θ has little influence on τ_{95}. As shown in fig. B1, **C**, the local dynamics of mixing are not very well captured by the CM, even if τ_{95} is comparable - the relatively large volume of compartments, modeled as ideally mixed zones, inherently induce some 'numerical blending' which affect the resolution of local dynamics. This is a conclusion similar to that by Delafosse et al. (Delafosse et al., 2014), even though in their 2 impeller setup, local dynamics were less pronounced to begin with, due to the resistance towards mixing being in the inter-compartment region (Haringa, Vandewijer, & Mudde, 2018b,a).

Appendix A.2. Dual impeller geometry

The two-impeller geometry is equally based on Jahoda et al. (Jahoda et al., 2007): $T = 0.29$ m, $H = 2T$, $C = T/3$, $\Delta C = T$ and $D = T/3$. The CFD setup equals that of the single impeller case, except only 180° of the domain is modeled, with a periodic boundary condition. In this case,the monitor point was placed $r = 9.5T/20, Y = T/4, \theta = 45^o$ and a tracer pulse was injected at $t = 0$ at $r = T/4, Y = 0.57$ m$, \theta = 225^o$, yielding the mixing curve reported in fig. B1, **D**.

As for the single impeller, a wide range of compartment resolutions is employed: $N_{ax} = 12 - 96$, $N_r = 2 - 12$, $N_\theta = 3 - 18$. N_{ax} is chosen

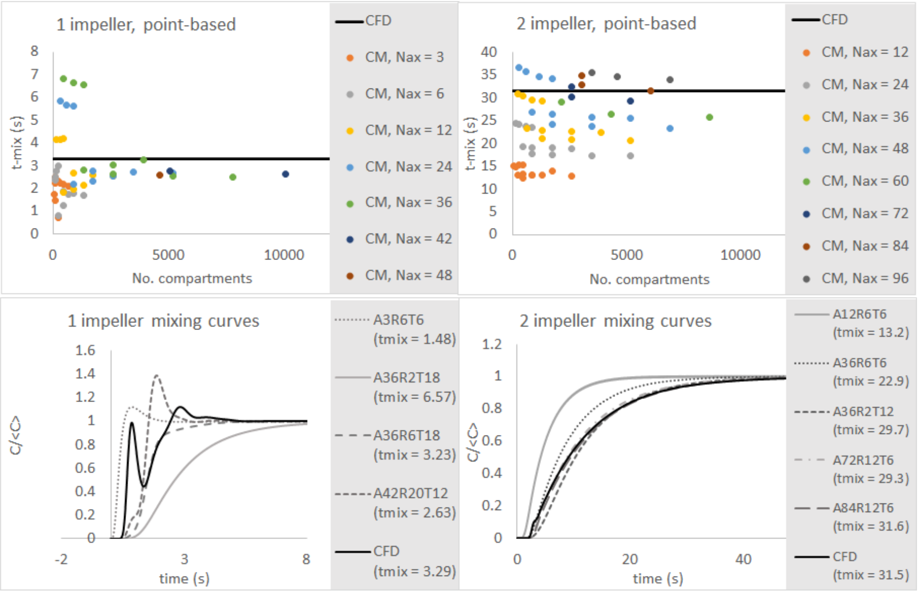

Figure B1: **A:** Mixing time (monitor point) for the 1-impeller setup of Jahoda; CFD versus various compartment realizations. **B:** As A, for the 2 impeller setup. **C:** Mixing curves for selected compartment realizations vs. CFD, 1 impeller. **D:** As C, for the 2 impeller setup.

such that the impellers and inter-impeller midplane, where fully radial flow is anticipated, consistently align with compartment interfaces. The same applies to the impeller blades (3 blades in the 180° domain) with respect to tangential divisions. Fig. B1, **B** shows the 95% mixing time, with symbols colored by the number of axial compartments. The impact of N_{ax} is even more pronounced than for the single impeller case, and as such, a wider scatter in τ_{95} vs. N_c is observed. This scatter may seem surprising, considering the good agreement observed by Delafosse et al. (Delafosse et al., 2014), but it must be kept in mind all their simulations featured high N_c $(4360 - 17600)$, whereas we explore much lower resolutions. With $N_{ax} > 60$, we observe results consistent with Delafosse. As before, a low N_r leads to a higher τ_{95}, although this effect is less pronounced, because the effect of low N_{ax} tends to dominate. Still, a proper 'balance' of N_{ax} and N_r can be used to yield a good prediction of τ_{95} at relatively low

N_C. For example, $36 \cdot 2 \cdot 12$ compartments leads to a good prediction of τ_{95}, including local dynamics that are well in line with the CFD result (fig. B1, D), because the impact of under-resolved axial and radial flow balances out. In general, the qualitative agreement between the mixing curves from CM and CFD (fig. B1, D) at the bottom of the reactor is much better for the 2-impeller case, due to the resistance towards mixing being in the inter-impeller region, leading to more gradual mixing in the bottom region which lacks the sharp oscillations observed in the 1-impeller case. This qualitative agreement is also well in line with the observations of Delafosse et al. (Delafosse et al., 2014).

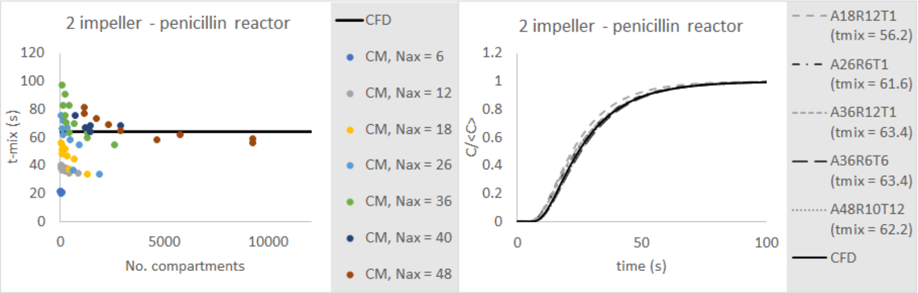

Figure B2: **A:** Mixing time (monitor point) for the penicillin reactor; CFD versus various compartment realizations. **B:** Mixing curves for selected compartment realizations vs. CFD.

Appendix A.3. Penicillin reactor

The penicillin reactor is also a 2-Rushton impeller geometry, although it differs somewhat from Jahoda et al. Most notably, the bottom impeller has 8 rather than 6 blades. The tank dimensions are $H = 7.7$ m, $C = T/3$, $\Delta C = T$ and $D = T/3$. The CFD setup used in this study is described in detail in earlier work (Haringa et al., 2016). Considering both τ_{95} versus N_C (fig. B2, **A**), and the mixing curves (fig. B2, **B**) for selected cases the trend is very similar to the Jahoda 2-impeller setup. As noted previously, this implies good results for τ_{95} can be acquired with relatively few compartments, provided the error induced by under-resolving N_{ax} and

N_r balances. While this solution is not 'grid-independent', it may be a pragmatic way to reduce the required number of compartments (hence computation time) in compartment model simulations.

Appendix A.4. Liquid-phase mixing performance: conclusions

Overall, we observe that for a sufficient number of compartments (typically N_c approaching 10^4) the mixing behavior in the CM is close to that of a full CFD simulation, and becomes independent of N_c. At high N_c, the agreement becomes comparable to the observations of Delafosse et al. (Delafosse et al., 2014), although in contrast to their work, we did not explore $N_c > 10000$, considering such compartment numbers would be impractical in later metabolic simulations. For a low number of compartments, there is substantial scatter in τ_{95} versus N_c, with under-resolution in the axial direction (low N_{ax}) leading to lower τ_{95} due to faster axial transport, and under-resolution in the radial direction (low N_r) leading to higher τ_{95} due to poor representation of the circulation flow. Properly setting the combination of N_r and N_{ax} can lead to a good prediction of τ_{95} at low N_c, which may facilitate metabolic computations by keeping the computational burden low. This result is not 'grid independent' - a poor selection of N_r and N_{ax} can easily lead to erroneous predictions in both mixing and metabolic response (more on this in Appendix B), and hence mixing performance should be validated against the CFD data. Additionally, it is recommended to verify the metabolic computations against a case with equal τ_{95} but higher N_C - even at equal τ_{95}, spatial under-resolution of the substrate gradient may affect the model output, which should be avoided.

Appendix A.5. Parcel mixing

As shown in the main text, for high N_p the Eulerian and Lagrangian mixing behavior are in good agreement. The timestep used in positional

updating can have an impact on the accuracy of the Lagrangian trajectories, however. In this work, the position is updated during each function call of the integrator (which is multiple times per timestep in the multistep methods employed here). If the Δt between two function evaluations is large, this will introduce inaccuracies in parcel behavior by introducing bias towards longer residence times. This effect is shown in figure B3, **A**. Here, simulations were conducted with various values for Δt_{max}. In addition, the Eulerian tracer concentration was set to 0 everywhere, so that all differentials dN/dt were equally zero; this results in the adaptive timestep solver (and hence parcel position updating) defaulting to the maximum stepsize. The impact of the introduced bias is clearly visible: mixing slows down compared to the Eulerian approach, and a plateau is reached at a higher CoM-value, indicating that the parcel distribution remains heterogeneous. Figure B3 **B** shows the same cases, but in this case the solution is simultaneously computing an Eulerian and Lagrangian mixing problem. In this case, the adaptive timestepping kicks in to control the accuracy of the Eulerian approach; the timesteps become sufficiently small to resolve mixing both in the Eulerian and Lagrangian approach (although statistical fluctuations in local N_p still lead to a finite plateau-CoM). These results indicate that as long as Eulerian dynamics are present, the adaptive timestep algorithm is sufficient to provide sufficiently accurate Lagrangian mixing, too. Conceptually, this is logical, considering that the physical mixing timescales for the Eulerian and Lagrangian phase are similar. However, in cases where Eulerian and Lagrangian behavior are decoupled, additional care may be needed to properly set the particle position update interval. As a first estimate, $\Delta << \tau_{res}$ seems a reasonable choice, with τ_{res} the residence time in compartment with the shortest residence time. For the cases tested in this work, the parcel distribution is reasonably homogeneous with $\Delta t_{max} = 0.03$, although, as will be noted in sec. Appendix B.2, some offset between the mean volumetric substrate concentration and mean substrate concentration registered by parcels is

still observed. This indicates there is still some heterogeneity in the parcel distribution; with $\Delta t_{max} = 0.001$ this difference is no longer observed, albeit at much increased computation time. As such, it is concluded the offset is a consequence of the finite timestep size, and not an inherent issue with the parcel tracking algorithm. It is up to the user to trade-off accuracy and computation time, for which we deemed $\Delta t_{max} = 0.03$ in the current work.

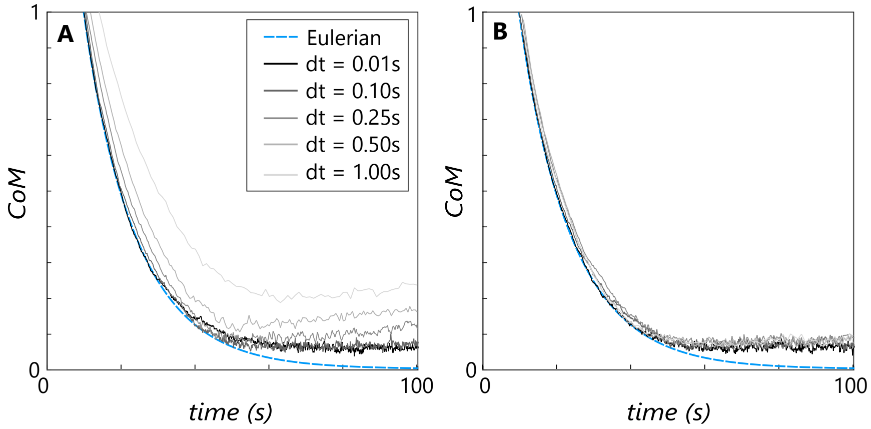

Figure B3: Lagrangian mixing behavior with $N_p = 25000$, $A18R2$, for various Δt. **A:** No timestep control. **B:** Timestep control by the Eulerian phase.

$$CoM(t) = \sqrt{\left(\frac{\Sigma_n(\frac{C_{n,t}-\overline{C}}{\overline{C}})^2 \Delta V_n}{\Sigma_n \Delta V_n}\right)} \tag{A.1}$$

Appendix B. Impact of various CM parameters on metabolic predictions and computation time

As a base case, we take $N_{ax} = 26$, $N_r = 6$, $N_\theta = 1$, $N_p = 1000$, $\Delta t =$ 0.03 s and integration relative tolerance $RelTol = 1E-3$. These settings were chosen for the manageable amount of compartments ($N_C = 156$), parcels per compartment (ca. 6.5) and solution time (ca. $1-1.5$ h for 80 h

flow time). Unless otherwise mentioned, parameter set 'TU-B' ($q_{s,max}$ = 1.6 mmol/g$_{dw}$/h and $K_s = 7.8\mu$mol/kg) is used.

All simulations reported in this work were conducted on a desktop computer with an INTEL i5-4460 (3.2GHz, quadcore) with 16GB 1600MHz DDR3 RAM, colloquially known as "a decent midrange gaming PC built on a PhD budget in 2015". The ratings presented below are the time required to solve the ODE-section of the code: pre-processing (memory allocation, parcel initialization and preparing the property vectors and transition matrices) and post-processing (data storage and/or plotting) are excluded. Pre-processing was found to be a minor component of the total time requirement, whereas the time for plotting depends very strongly on the number of datapoints plotted, rather than the size of the system itself. All numbers were treated as Float64 (double-precision)

The current Julia code was constructed following the efficient computation guidelines offered in the DifferentialEquations.jl documentation, keeping into account the following:

- In-place computations were used to avoid allocation of memory during computation (updating the system of equations and parcel positions required 0 allocations). All arrays were pre-allocated, and no global variables were used.
- The equations were devectorized into for-loops to yield minimal overhead in the compiled code.
- Recurring mathematical operations on constants were pre-computed (e.g. recurring divisions or exponents).
- Considering the inherent fluctuations in the solution, a high numerical accuracy was not deemed necessary: the BS3 solver was used, for offering the highest speed. A comparison with the TSIT5 solver (default) was made.

Appendix B.1. Prediction of q_p in comparison to full CFD

As observed in sec. 3.3 of the main article, it was noted that with Lagrangian reaction coupling, the relative offset in q_p between full CFD and CM (for parameter set 'TU-B') is ca. 10%. This discrepancy mainly originated from parcels in the CM being more exposed to high substrate concentrations, evident from the larger excess-zone and higher mean substrate concentration $\overline{C_{s,p}}$ (sec. 3.2), which, through higher glycolytic intermediates, significantly impacts the prediction for q_p (Appendix D). As $\overline{C_{s,p}}$ is higher than the full CFD simulation for all reported values of N_p as well as for the Eulerian simulation with black-box kinetics reported in the main article, it appears the offset is caused by the notion that the tested compartment layout does not fully represent the hydrodynamics of the full CFD simulation, which is unsurprising as only the single-point mixing time prediction was used as a matching criterion. As such, we expect the q_p offset of ca. 10% is a case-specific observation rather than due to an inherent limitation with the simulation approach, and that a compartment layout that better represents the C_s distribution in the full CFD simulator will yield better agreement in q_p. For typical engineering applications, we deem the current level of accuracy sufficient.

Appendix B.2. Impact of N_p

Using the base case for all other settings, N_p is varied between $50 - 2500$. With $N_{pool} = 8$ dynamic pools, the total number of equations in the system is $N_c + N_p \cdot N_{pool}$. If we plot τ_{run} versus the total number of equations, the time requirement scales linear (fig. C 2) - hence an increase in N_p gives a proportional increase in τ_{run}. The results are quantitatively summarized in table C 2 for A26R6T1. This table also list the impact on the parameter of interest in this study, q_p, at the end of the 80 h simulation timeframe. It can be observed that in all cases, the q_p in the compartment model is lower than in the CFD simulation - up to 10 % relative offset compared

to the CFD simulation for case TU-B. A weak impact of the number of parcels N_p can be observed, with the relative offset compared to full CFD asymptotically increasing to ca. 10.5 % for large N_p. Similar behavior was observed in full CFD simulations with low N_p (fig. 5 in (Haringa et al., 2017)). The reason for this is that with Lagrangian coupling, substrate uptake is concentrated in the compartments containing parcels, which with low N_p will only be a fraction of the compartments. Consequently, C_s in the parcel-containing compartments is reduced, but in the others, without parcels, it will increase; overall, the average volumetric concentration $\overline{C_s}$ will rise. This is indeed observed in table 1 of the main article, can be deduced from the compartment concentration traces in figure 4 of the main article, and is reported here in fig C 1, **A**. However, as parcels are in compartments where, due to their presence, uptake is always active, they will see a *lower* average substrate concentration, $\overline{C_{s,p}}$ (fig. C 1, **B**), with the discrepancy between $\overline{C_s}$ and $\overline{C_{s,p}}$ being large for low N_p, and reducing for large N_p. As can be observed in fig. C 1, **B**, for $N_p \to \infty$, the Eulerian limit is not fully approached, and there is a residual offset between $\overline{C_s}$ and $\overline{C_{s,p}}$. In table C 1 it is also visible that, for case A26R6T1-Euler, there is an offset between $\overline{C_s}$ and $\overline{C_{s,p}}$ still. This is attributed to a imperfect distribution of the parcels for $\Delta t = 0.03$ s; sec. Appendix A.5 shows that a large timestep size introduces bias in the parcel distribution; for $\Delta t = 0.03$ s the distribution is nearly but not completely homogeneous. This is supported by a short Eulerian-mode simulation. spanning $t = 3600$ s, discarding the dynamic first 100 s while averaging, with $N_p = 1000$ and $\Delta t = 0.001$ s yielding $\overline{C_s} = 39.2 \cdot 10^{-6}$ and $\overline{C_{s,p}} = 39.3 \cdot 10^{-6}$ mol/kg. Hence, with small enough timesteps the discrepancy is fully removed, but the runtime becomes infeasible.

The production rate q_p is linked to variations in C_s, and a higher mean concentration is associated with stronger substrate heterogeneity (Haringa et al., 2016)/stronger excess substrate conditions leading to a lower q_p. As

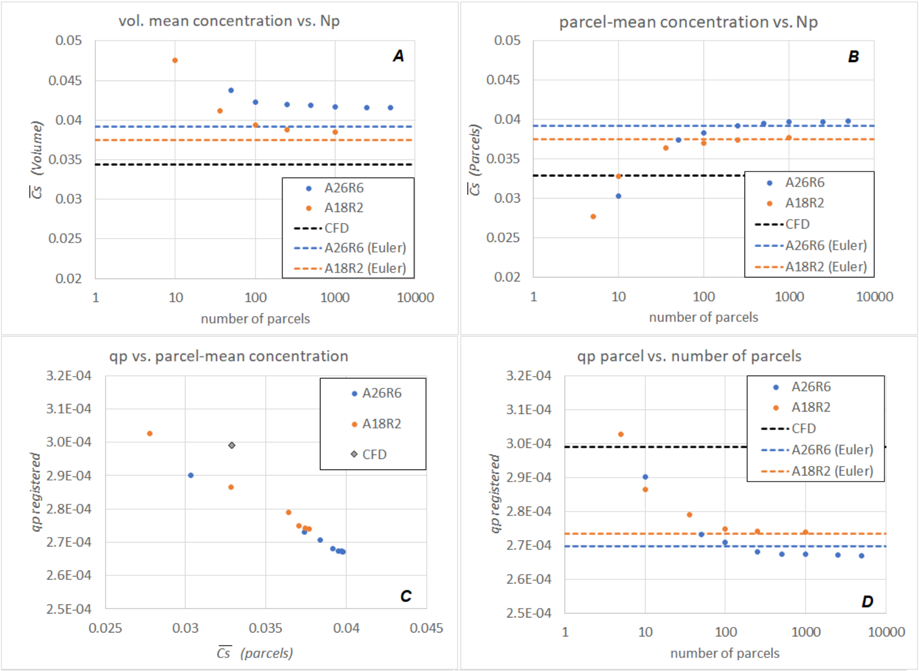

Figure C 1: Impact of the number of parcels N_p on the observed mean concentration and penicillin production rate q_p for two compartment layouts. **A:** Mean volumetric substrate concentration $\overline{C_s}$ (mol/kg), as a function of N_p. **B:** Mean substrate concentration $\overline{C_{s,p}}$ (mol/kg) observed by parcel population, as a function of N_p. **C:** Registered final penicillin production rate q_p (mol/Cmol/h) as a function of the parcel-mean substrate concentration $\overline{C_{s,p}}$. **D:** Registered final penicillin production rate q_p (mol/Cmol/h) as a function of N_p.

the metabolic model is linked to the parcels, $\overline{C_{s,p}}$ should be considered. Indeed, in fig C 1, **C**, a higher $\overline{C_{s,p}}$ correlates strongly with lower q_p. The trend for the compartment model does deviate somewhat from full CFD indicating that the C_s-distribution in the compartment model differs somewhat from the full CFD; for example, table C 1 shows that even with a lower $\overline{C_{s,p}}$, the excess exposure experienced by parcels in case $A18R2$ with $N_p = 10$ is still larger than in full CFD, and the large impact of high C_s still leads to a lower q_p-prediction compared to full CFD. At increasing N_p, both $\overline{C_{s,p}}$ and q_p converge to a limit value, which nearly equals the result obtained when releasing parcels in a concentration field obtained with Eulerian reaction coupling, thus uncoupling the concentration gradient from the parcel positions (fig. C 1 , **D**). This limit is approached rapidly, showing that even with limited numbers of parcels (N_p 100), an approximation reasonably independent of N_p is provided. Overall, from fig. C 1, we can see that for most situations $\overline{C_{s,p}} > \overline{C_{s,CFD}}$. This, combined with a substrate concentration distribution skewed towards excess in comparison with CFD (table C 1), explains why the compartment model in the majority of tested cases leads to lower predictions for q_p than the full CFD simulation for parameter set 'TU-B'.

When it comes to time requirements, for different compartment layouts (A6R6T1 and A36R6T1) a linear impact is observed. Table C 3 lists the time requirement for the other cases.

Appendix B.3. Impact of N_c

In fig. C 2, **A**, the gray symbols indicate the computation time for a fixed $N_p = 1000$ and a varying number of compartments (details in table C 4). Here, a strong impact of N_C on the runtime is observed, scaling with $\tau_{run} \approx N_c^{3.9}$ in the studied range. There are a number of factors leading to this sharp increase: N_c compartments have N_c^2 potential connections, increasing the computation time of the flow equations. In addition, the

Table C 1: Impact of N_p on the mean concentration (volumetric and parcel-based, in mol/kg), regime distribution (parcel-based) and q_p (mol/Cmol$_x$/h). For the Euler-simulations, the concentration gradient was computed with Eulerian reaction coupling (sec. 3.2 of main article), with 100 parcels reading the concentration in their surroundings and computing their response on that reading. Other settings: $\Delta t = 0.03$ s, RelTol = 0.001.

Comp/N_p	$\overline{C_s}$	$\overline{C_{s,p}}$	$E/L/S$ (field)	$E/L/S$ (parcel)	q_p
CFD	$34.4 \cdot 10^{-6}$	$34.4 \cdot 10^{-6}$	6.8/36.2/57.0	6.8/36.2/57.0	$2.99 \cdot 10^{-4}$
$A26R6T1/Euler$	$39.2 \cdot 10^{-6}$	$38.1 \cdot 10^{-6}$	8.7/33.9/57.4	–	$2.65 \cdot 10^{-4}$
$A26R6T1/10$	$56.0 \cdot 10^{-6}$	$30.3 \cdot 10^{-6}$	12.7/57.3/30.0	6.8/47.3/45.9	$2.90 \cdot 10^{-4}$
$A26R6T1/1000$	$41.7 \cdot 10^{-6}$	$39.7 \cdot 10^{-6}$	9.6/36.4/54.0	9.1/35.7/55.2	$2.67 \cdot 10^{-4}$
$A18R2T/Euler$	$37.5 \cdot 10^{-6}$	$37.1 \cdot 10^{-6}$	10.4/31.4/58.2	–	$2.74 \cdot 10^{-4}$
$A18R2T1/10$	$47.5 \cdot 10^{-6}$	$32.8 \cdot 10^{-6}$	12.0/42.1/45.9	8.4/39.8/51.8	$2.87 \cdot 10^{-4}$
$A18R2T1/1000$	$38.5 \cdot 10^{-6}$	$37.7 \cdot 10^{-6}$	10.4/33.6/56.0	7.8/9.8/51.8	$2.74 \cdot 10^{-4}$

Table C 2: Impact of N_p on runtime τ_{run}. Other settings: $\Delta t = 0.03$ s, RelTol = 0.001.

Compartments	parcels	τ_{run} $[s]$	$q_p(end)$	$\%\ diff.\ CFD$
$A26R6T1$	50	640	$2.73 \cdot 10^{-4}$	-8.6
$A26R6T1$	100	850	$2.71 \cdot 10^{-4}$	-9.3
$A26R6T1$	250	1236	$2.68 \cdot 10^{-4}$	-10.2
$A26R6T1$	1000	4247	$2.67 \cdot 10^{-4}$	-10.5
$A26R6T1$	2500	10783	$2.67 \cdot 10^{-4}$	-10.6
$A26R6T1$	5000	21585	$2.67 \cdot 10^{-4}$	-10.6
$A26R6T1$	10-Eulerian	171	$2.65 \cdot 10^{-4}$	-11.3

Table C 3: Impact of N_p on runtime τ_{run}, with different compartment numbers. Other settings: $\Delta t = 0.03$ s, RelTol = 0.001.

Compartments	N_c	N_p	τ_{run} $[s]$
$A6R6T1$	36	250	1026
$A6R6T1$	36	1000	4089
$A6R6T1$	36	2500	10109
$A36R12T1$	432	250	3519
$A36R12T1$	432	1000	8599
$A36R12T1$	432	2500	21563

lower residence time per compartment as well as lower number of parcels per compartment (leading to larger fluctuations in local biomass concentration) increase the stiffness of the problem - requiring smaller timesteps to properly resolve parcel behavior.

As observed in table C 4, q_p differs substantially from that computed in the CFD simulation for cases A6R6T1 and A36R2T1. This offset is easy to explain: the mixing time in A6R6T1 is strongly under-estimated compared to CFD, while for A36R2T1 it is substantially over-estimated. This hence leads to a substantial under-estimation in extracellular heterogeneity for the former, and over-estimation for the latter, which affect q_p. For all the other cases, the mixing time is close to the CFD result ($\tau_{95} = 63.8$). For these cases, with the current kinetic parameters ($q_{s,max} = 0.0016$ mol/g$_{dw}$/h and $K_s = 7.8 \cdot 10^{-6}$ mol/kg), the offset compared to full CFD differs case-by-case, but typically lies between 5 and 10 % relative offset. As discussed in sec. Appendix B.2, the reason is that the compartment model C_s distribution typically features a somewhat larger excess-zone and higher $\overline{C_{s,p}}$ at equal τ_{95}, leading to a somewhat lower q_p.

Here, it's important to note that we currently aimed at matching mixing time based on a single measurement point. It can be that a more thorough

assessment provides other criteria for determining what the most representative compartment layout is (for example, matching the C_s-distribution in the Eulerian formulation, or a multi-point/volumetric mixing comparison), and that such layouts lead to better matched predictions between CFD and CM in terms of q_p. This is a subject for further work.

Table C 4: Impact of N_c on runtime τ_{run}. Other settings $N_p = 1000$, $\Delta t = 0.03$ s, RelTol = 0.001.

Compartments	N_c	τ_{run} $[s]$	τ_{95} $[s]$	$q_p(end)$	$\%\ diff.\ CFD$
$A6R6T1$	36	4089	20.3	$3.68 \cdot 10^{-4}$	23.2
$A18R2T1$	36	3974	56.2	$2.74 \cdot 10^{-4}$	−8.3
$A36R2T1$	72	4164	97.2	$2.26 \cdot 10^{-4}$	−24.4
$A26R6T1$	156	4247	61.6	$2.67 \cdot 10^{-4}$	−10.5
$A36R12T1$	432	8598	63.4	$2.78 \cdot 10^{-4}$	−6.8
$A36R6T6$	1296	9866	63.3	$2.72 \cdot 10^{-4}$	−8.9
$A48R10T6$	2880	29602	65.1	$2.78 \cdot 10^{-4}$	−6.9
$A48R10T12$	5760	87252	62.2	$2.85 \cdot 10^{-4}$	−4.7

Appendix B.4. Impact of Δt_{max}

Fig. C 2, **B** shows the impact of Δt_{max} for the base case setup. Note that we are using a variable timestep solver, so in order to control accuracy, the used timestep may be smaller than Δt_{max}. This is clearly reflected in the figure: for $\Delta t_{max} > 0.06$ s the runtime τ_{run} is constant, indicating the maximum timestep size is not used. Conversely, the computation time for $\Delta t_{max} = 0.015$ s is double that of $\Delta t_{max} = 0.03$ s, which indicated the maximum timestep size *is* limiting the computation here (and the runtime scales inversely with Δt_{max} in this range). As mentioned in section Appendix B.3, the 'optimal' Δt likely depends on N_c because the compartment residence time decreases with an increase in compartment number, and smaller steps are needed to properly resolve parcel behavior.

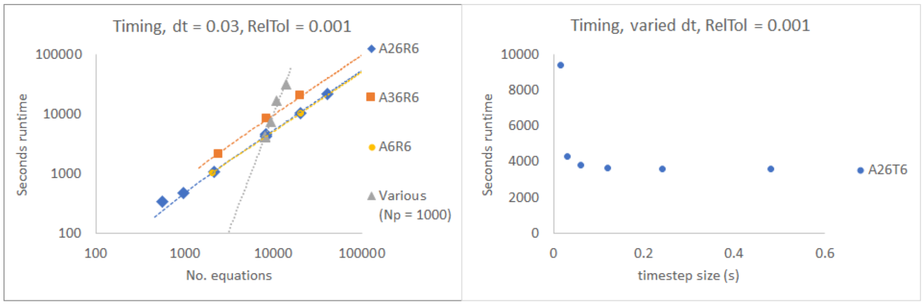

Figure C 2: Impact of various parameters on computation time. **Left:** the computation time as a function of the number of equations (parcel*pools + compartment), for 3 compartment layouts and a variable number of parcels Np. The scaling with $N_p \cdot N_{pool}$ is linear. The gray curve shows a fixed number of parcels ($N_p = 1000$), with a variable number of compartments. Scaling follows a power law N_c^n with $n > 3$, showing an increase in compartments has a large impact on computational burden. **Right:** The impact of maximum timestep size Δt on the result. For the given settings, the results indicate the maximum timestep size is not used for $\Delta t > 0.06$, hence the computation time is independent of Δt.

The results are quantitatively summarized in table C 5. Considering the agreement for q_p, a higher Δt_{max} seems to lead to a very slightly lower q_p, because there is some bias towards longer compartment residence times (hence a slight increase in parcel circulation time). However, the offset in q_p effectively remains constant, indicating the use of an adaptive timestepping algorithm prevents a residence time bias once metabolic coupling is introduced, meaning manual tuning of Δt_{max} is not required.

Table C 5: Impact of Δt on runtime τ_{run}

Compartments	Δt_{max} $[s]$	τ_{run} $[s]$	$q_p(end)$	$\% \ diff. \ CFD$
$A26R6T1$	0.015	9582	$2.69 \cdot 10^{-4}$	-10.0
$A26R6T1$	0.03	4247	$2.67 \cdot 10^{-4}$	-10.5
$A26R6T1$	0.06	3430	$2.66 \cdot 10^{-4}$	-10.9
$A26R6T1$	0.12	3462	$2.66 \cdot 10^{-4}$	-10.8
$A26R6T1$	0.24	3397	$2.66 \cdot 10^{-4}$	-10.9
$A26R6T1$	0.48	3506	$2.66 \cdot 10^{-4}$	-10.8

Appendix B.5. Impact of RelTol

Interestingly, RelTol has no large impact the solution accuracy, or the computation time (table C 6) , which may appear to contradict the statement in the previous segment. Most likely, this is because the pool dynamics are slow in comparison to $\Delta t_{max} = 0.03$ s (even with the rapid extra-cellular uptake of the current microbe, $\tau_{rxn} \approx 12\Delta t_{max}$. As long as a parcel remains in place, tolerances are easily met. The situation changes if parcels are moving around, which causes discontinuities in the uptake terms due to sudden changes in the observed substrate concentration, $C_S(p)$. Hence, we hypothesize that the previously noted impact on Δt results from the frequency of parcel jumps and consequent rapid changes in uptake (as, in this case, the Lagrangian and Eulerian frameworks are coupled), and not from the solver tolerance.

Table C 6: Impact of tolerance on runtime τ_{run}

Compartments	$RelTol$	τ_{run} $[s]$	$q_p(end)$	$\%\ diff.\ CFD$
$A26R6T1$	$1E-2$	4497	$2.67 \cdot 10^{-4}$	-10.5
$A26R6T1$	$1E-3$	4247	$2.67 \cdot 10^{-4}$	-10.5
$A26R6T1$	$1E-4$	4650	$2.67 \cdot 10^{-4}$	-10.5

Appendix B.6. Impact of Solver

All simulations were conducted with the BS3 algorithm, based on Julia benchmarks for ODE solutions with high RelTol. We compared the result to the default algorithm for differential equations - Tsit5. The difference in runtime between these algorithms is approximately a factor 2 (table C 7), without a notable difference in q_p.

Appendix B.7. Impact of kinetic parameters

As was mentioned in the main article, simulations conducted with the parameters from case 'TU-A', $q_{s,max} = 0.00113$ /g$_{dw}$/h and $K_s =$

Table C 7: Impact of tolerance on runtime τ_{run}

Compartments	solver	τ_{run} $[s]$	$q_p(end)$	$\%\ diff.\ CFD$
$A26R6T1$	Tsit5	8066	$2.67 \cdot 10^{-4}$	-10.6
$A26R6T1$	BS3	4247	$2.67 \cdot 10^{-4}$	-10.5

$9.8 \cdot 10^{-6}$ mol/kg, based on the metabolic model parameterization of (Tang et al., 2017), showed good agreement in terms of q_p, despite being based on the same hydrodynamic compartment model. As can be observed in table C 8, this is not due to a better agreement in the mean concentration $\overline{C_{s,p}}$ or the excess/limitation/starvation distribution, which, as for case 'TU-B', are skewed towards excess concentrations. As such, the results indicate that for case 'TU-A' the more pronounced negative impact of excess concentrations is balanced out by the less pronounced negative impact of starvation conditions, whereas for 'TU-B', the two do not balance. To exclude the possibility of a parameterization or implementation error in the kinetic model, lifelines acquired in the CM for both parameter sets have been exported to *.csv* files which were subsequently loaded in MATLAB 2021b, and scrutinized with the kinetic model implementation of (Haringa, Tang, et al., 2018). The observations were a perfect match between MATLAB and the current CM, thus validating the kinetic model implementation.

Appendix B.8. Metabolic predictions and timing: conclusions

Overall, the factors that have the biggest impact on the solution timing are N_c and N_p. In particular N_C has a substantial impact on the overall computation time, probably because increasing N_C both leads to a larger system of fluid equations, and because finer timesteps are used by the adaptive timestepper in order to properly resolve parcel behavior (due to the shorter per-compartment residence time). For the studied range of compartment numbers, the predicted q_p was negligibly influenced by N_C

Table C 8: Mean parcel-based concentration $\overline{C_{s,p}}$ (mol/kg), regime distribution, predicted q_p (mol/Cmol$_x$/h) and relative offset compared to CFD with the kinetic parameters of TU-A.

Comp/N_p	$\overline{C_{s,p}}$	$E/L/S$ (parcel)	q_p	$\%\ diff.\ CFD$
CFD	$57.0 \cdot 10^{-6}$	10.3/50.5/39.2	$3.57 \cdot 10^{-4}$	–
$A26R6T1/Euler$	$61.3 \cdot 10^{-6}$	13.3/51.8/34.9	$3.52 \cdot 10^{-4}$	−1.3 %
$A26R6T1/1000$	$64.7 \cdot 10^{-6}$	14.0/51.7/34.3	$3.54 \cdot 10^{-4}$	−0.8 %
$A26R6T1/2500$	$64.8 \cdot 10^{-6}$	14.1/51.6/34.3	$3.54 \cdot 10^{-4}$	−0.8 %
$A18R2T1/Euler$	$61.7 \cdot 10^{-6}$	15.6/49.0/35.4	$3.54 \cdot 10^{-4}$	−1.0 %
$A18R2T1/36$	$60.0 \cdot 10^{-6}$	12.8/52.3/34.9	$3.54 \cdot 10^{-4}$	−0.8 %
$A18R2T1/1000$	$61.6 \cdot 10^{-6}$	14.3/51.0/34.7	$3.54 \cdot 10^{-4}$	−0.8 %

compared to the full-CFD result, provided the mixing time matched that of the CFD simulation. The number of parcels N_p has a linear impact on the computation time in the studied range. Interestingly, the agreement in q_p was hardly impacted by N_p, even for just 50 parcels in 156 compartments - meaning only 1 in 3 compartments even contain a single parcel at any time, and C_s in each compartment is quite variable. This is likely because the timescales of the intra-cellular pools, in particular q_p, are very long compared to the timescale of the (artificial) fluctuations induced by the under-resolved biomass phase. Hence, the impact of these spurious fluctuations is buffered. Note this conclusion may not generalize to other systems, the impact of N_p should be studied on a case-to-case basis, and with respect to the parameter of interest. In addition, note that currently only the mean pool size was compared between the CFD simulation and CM simulation, and it was not checked whether the number of parcels was adequate to fully represent the population distribution of the pool (i.e. if the higher order moments were also matched). It may be that a larger N_p is needed to ensure the full pool distribution is independent of N_p, and as such, to draw conclusions on the emergence and distribution of population heterogeneity.

In this particular case q_p is the parameter of interest, and it is reasonably well predicted even for a low N_c and N_p, using the matching criterion that the single-point τ_{95} is close to the CFD simulation. The results show that for low N_c and N_p a simulation of 80 h flow time can be conducted in several minutes - compared to weeks for a full CFD simulation with frozen flow. The q_p offset that is noted seems to be a case-dependent observation due to some degree of over-estimation of the excess zones in the reactor, not a structural offset related to the model implementation. As such, we expect that further investigation into the optimal compartment layout to match the full CFD simulation, possibly using automated compartment generation approaches, can improve the agreement between the CM and CFD. However, for typical engineering applications (quick assessment of the impact of substrate heterogeneity, crude scanning of a design space), we deem the current accuracy to suffice.

Appendix C. Additional notes on compartment flux calculations

In this appendix a few additional remarks are made with respect to the compartment generation algorithm as implemented in ANSYS FLUENT. As was mentioned in the article, the algorithm consists of the following steps:

1. Loop over all gridcells, and assign a compartment number n based on their spatial location. The collective of gridcells with the same compartment number n forms a single compartment N.
2. Loop over all gridcells and sum the cell volume for cells with the same n to get the total volume for compartment N. The same procedure can be applied to other relevant cell values, such as energy dissipation, pressure, etc., that may be required in the compartment model. In case the compartment average of a quantity is required, the volume- or mass-weighted sum can be collected, and divided afterwards by total volume or mass.

3. Loop over all gridcell faces. If a face lies on the compartment interface (i.e. the compartment number n is different between the left- and right-side of the face), calculate the convective through the face, and modify convective flow matrix Φ_c accordingly. The absolute flux value is added at location ϕ_{ji} where j is the compartment number the flux flows in to, and i the compartment number the flow flows out of, to account for the mass entering compartment j. Also subtract the same absolute value from location ϕ_{ii} to account for the withdrawal of the same mass from origin compartment i.
4. In the same face-loop, compute the turbulent flux from the turbulent kinetic energy at the cell face. Because turbulence is assumed to be of equal strength bidirectionally, equal values are added to locations $\phi_{T,ji}$ and $\phi_{T,ij}$ in turbulent flux matrix Φ_T, and subtracted from $\phi_{T,ii}$ and $\phi_{T,jj}$.

The convective fluxes are computed using the UDF macro F_Flux, which computes the mass flow rate through a face and returns a positive value if the flow is in the direction of the face area normal direction, and a negative number otherwise. The sign of the returned value is hence used to determine if the flux is directed from compartment i to compartment j, or from j to i.

Appendix C.1. Application of algorithm to different compartment maps

The setup of the flux determination is such that the flux is in the normal direction of the local gridcell-face area everywhere. In the current structured cylindrical compartment division algorithm, this practically leads to a set of orthogonal fluxes. However, this is not the case in general; several authors have proposed compartment generation algorithms that consider local flow features (Bezzo et al., 2003, 2004, Tajsoleiman et al., 2019, Nadal-Rey et al., 2021), leading to arbitrarily shaped compartments. In such cases, the fluxes are not orthogonal. In any case, the flux from compartment i to j reflects the total transport of mass in the i-to-j direction,

through all gridcell-faces that together make up the compartment interface between them, as illustrated in fig. C1

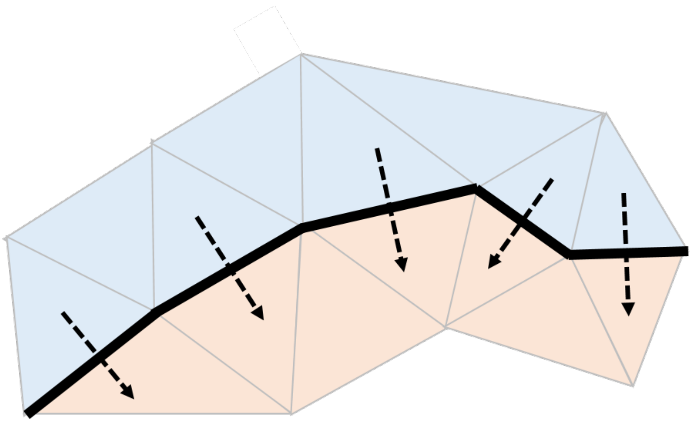

Figure C1: Illustration of the flux calculation between two curved compartments. The total flux between the compartments is the sum of the fluxes represented by the dashed arrows. At each gridcell-face, the direction of the flux is in the normal direction of the local interface.

The advantage of the flux calculation algorithm is that it is straightforward to implement, and places the compartment interface directly on the underlying CFD grid-faces, for which the velocity and k_t values are already available in the CFD solver, avoiding the need for additional interpolation based on cell-center values. The drawback of using the local face-normal fluxes is that, in some cases, unrealistically high fluxes between compartments are quantified. This happens can happen in two cases: (1) the CFD grid is not properly aligned with the flow direction and (2) the compartment grid is not properly aligned with flowfield features.

The first may, for example, occur if the interface between two compartments is rugged. This can be the case if a straight compartment interface is mapped on an unstructured grid, as illustrated in figure C2. In this example, the compartment interface is constructed such that it runs parallel to the flow direction, meaning that the total convective flux through this interface, ϕ_{ji} (and also in the converse direction, ϕ_{ij}) should be zero. With

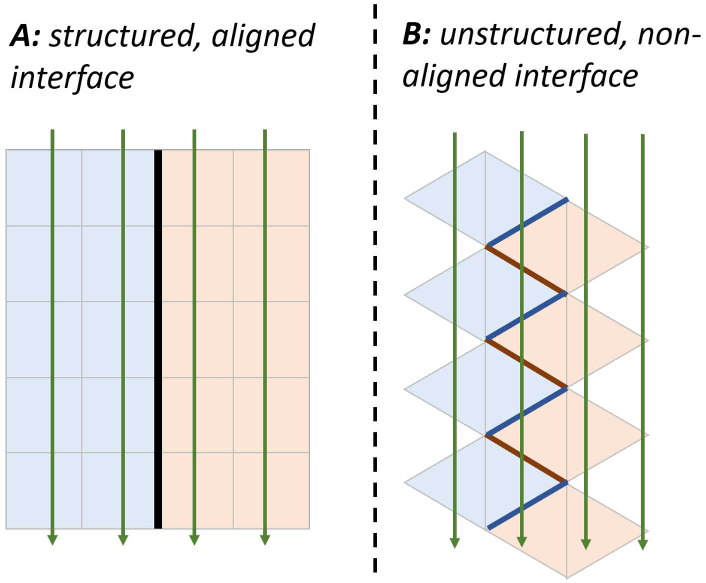

Figure C2: Illustration of potential flux over-estimation on rugged surfaces. In fig. **A**, the flow, structured CFD grid and compartment grid are aligned, leading to zero flux between the compartments. In fig. **B** the unstructured CFD-grid is not aligned with the flow, resulting in a rugged compartment interface through which the flux is non-zero (despite the compartment macroscopically being aligned with the flow direction.

an aligned rectangular grid, this (fig. C2, **A**), this will indeed be the case. However, if a non-aligned grid is used, as in C2, **B**, both ϕ_{ji} (the sum of face-flux f over all blue interfaces) and ϕ_{ij} (the sum of f over all red interfaces) will be non-zero. Hence, there will be artificial mixing between compartments i and j, which will affect mixing behavior in the overall compartment model. In this case, it may be a better option to decompose the face-fluxes into the component orthogonal to the overall *compartment interface*, rather than the *local gridcell-face*. An alternative would be to first interpolate the velocity data on a fine, structured grid, and then define the compartments on this interpolated data, as was done by others (Tajsoleiman et al., 2019).

The second example happens, for example, if the compartment layout is such that segregating features in the flowfield (e.g. regions of radial flow generated by Rushton turbines) lie within a single compartment, the assumption that the interior of a compartment is ideally mixed will undo the detrimental effect that such flow regions have on mixing (Haringa, Vandewijer, & Mudde, 2018a,b), as illustrated in figure C3. To avoid this issue, the compartment layout should line up with flow features. In this case, it was done by choosing the number of axial divisions such that the compartment interfaces lined up with relevant flow features. Others have achieved the same by manually specifying interfaces (Spann, Gernaey, & Sin, 2019), but for complex flowfields, the most robust option is to use feature-based compartment generation (Tajsoleiman et al., 2019), for example grouping compartments based on direction and magnitude of the axial velocity. Regardless of which option of compartment generation is preferred, all are compatible with the Euler-Lagrange compartment model presented in this study, as the exchange of both fluid and particles between compartments is solely dependent on the fluxes between neighboring compartments, and the compartment volumes.

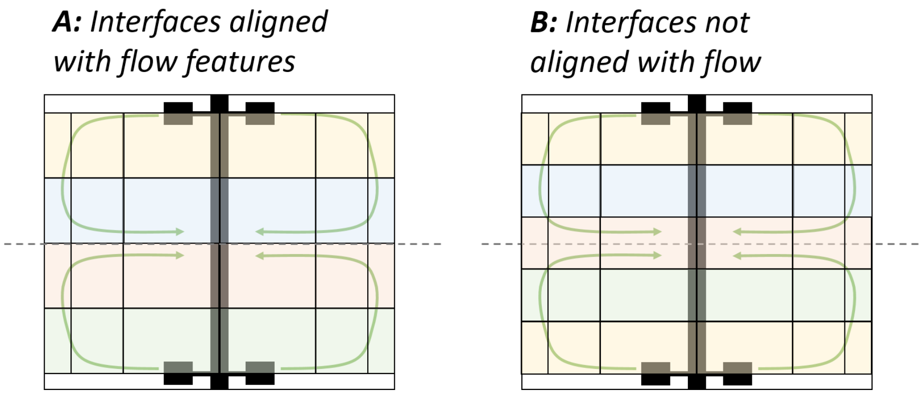

Figure C3: Illustration of mixing over-estimation due to misalignment between compartment layout and flow structure. The radial flow in the mid-compartment plane (dashed line) forms a barrier to mixing. **A:** Good alignment between the compartment layout and flow features in the inter-compartment region; mixing behavior from the CFD simulation is well represented. **B:** Poor alignment between compartment layout and flow features. Transport through the mid-compartment plane will be strongly over-estimated due to the ideal mixing assumption inside the compartments.

Appendix D. The 9-pool model for penicillin production

The 9-pool model developed by Tang et al. (Tang et al., 2017) is used to quantify the change in penicillin production in response to extra-cellular gradients. The stoichiometry is given in table C1, the model equations are summarized in table C2, and to conclude, the kinetic parameters are provided in C4. In full CFD simulations, it was noted that the ATP balance would diverge in response to rapid extra-cellular variations in glucose concentration (Haringa, Tang, et al., 2018). From a large range of simulated conditions, it was found that the intra-cellular ATP concentration (X_{ATP}) could reasonably be correlated with the concentration of intra-cellular glycolytic intermediates (Haringa, Tang, et al., 2018), thereby replacing the full ATP balance by an algebraic relation. We used the equation $X_{ATP} = A \cdot X_{gly}^3/(X_{gly}^3 + B^3)$, with $A = 8.5$ µmol/g$_{dw}$ and $B = 10.5$ µmol/g$_{dw}$.

Table C1: Stoichiometric matrix of the metabolically structured kinetic model (in mol/mol, from: Tang et al. (Tang et al., 2017)). X_{bio} is the biomass pool, *gluc* the extra-cellular glucose pool.

Pool	v_{11}	v_{12}	v_{13}	v_{21}	v_{22}	v_{31}	v_{32}	v_{33}	v_{41}	v_{42}	v_d
Glyc.	6	-1	-0.578	-1	0	0	0	-4.81	-1.07	1	0
AA.	0	1	-0.5	0	0	0	0	-6.25	0	0	0
Sto.	0	0	0	0	0	0	0	0	1	-1	
ATP	-2	-0.65	-1.037	4.43	-1	0	-2	-8	-0.167	-0.167	0
PAA	0	0	0	0	0	1	-1	-1	0	0	0
Gluc.	-1	0	0	0	0	0	0	0	0	0	0
X_{bio}	0	0	1	0	0	0	0	0	0	0	-1

Table C2: Kinetic equations of the $9-pool$ model.

Reaction	Kinetics	Eq.
Transporter capacity	$\frac{dX_{E,11}}{dt} = q_{E,11,max} \cdot \frac{((\mu+\mu_0)/k_{11})^5}{1+((\mu+\mu_0)/k_{11})^5} - (\mu + k_{dE,11})X_{E,11}$	$dv_{X,11}$
Carbon uptake	$v_{11} = k_{E,11}X_{E,11}\frac{C_s}{C_s+K_{s,11}}$	v_{11}
Amino Acid synthesis	$v_{12} = v_{12,max} \cdot \frac{X_{gly}^2}{K_{gly,12}^2+X_{gly}^2} \cdot \frac{K_{AA,12}^2}{K_{AA,12}^2+X_{AA}^2} \cdot \frac{X_{ATP}^3}{K_{ATP,12}^3+X_{ATP}^3}$	v_{12}
Growth	$v_{13} = v_{13,max} \cdot \frac{X_{gly}^2}{K_{gly,13}^2+X_{gly}^2} \cdot \frac{X_{AA}^2}{K_{AA,13}^2+X_{AA}^2} \cdot \frac{X_{ATP}^3}{K_{ATP,13}^3+X_{ATP}^3}$	v_{13}/μ
ATP production	$v_{21} = v_{21,max} \cdot \frac{X_{gly}^3}{K_{gly,21}^3+X_{gly}^3} \cdot \frac{K_{ATP,21}^4}{K_{ATP,21}^4+X_{ATP}^4}$	v_{21}
Maintenance	$v_{22} = m_{ATP,22}$	v_{22}
PAA import	$v_{31} = k_{perm,31} \cdot a_{cell} \cdot \left(\frac{X_{PAA}/2.5}{1+10^{pH,ext-pK}} - \frac{C_{PAA} \cdot \rho_{broth}}{1+10^{pH,int-pK}}\right)$	v_{31}
PAA export capacity	$\frac{dX_{E,32}}{dt} = \alpha_{32} + \beta_{32} \cdot \mu - k_{dE,32} \cdot X_{E,32} - \mu \cdot X_{E,32}$	$dv_{X,32}$
PAA export	$v_{32} = X_{E,32} \cdot X_{PAA} \cdot M_X \cdot 10^{-6}$	v_{32}
Pen-G production capacity	$\frac{dv_{33}}{dt} = \frac{\beta_{33} \cdot \mu}{1+(X_{gly}/K_{gly,33})^{m33}} - (k_{dE,33} + \mu) \cdot v_3 3$	v_{33}/q_p
Storage capacity	$\frac{dX_{E,4}}{dt} = \alpha_4 + \beta_4 \cdot \mu - k_{dE,4} \cdot X_{E,4} - \mu \cdot X_{E,4}$	$dv_{X,4}$
Carbon storage	$k_{41} \cdot X_{E,4} \cdot \frac{C_s}{C_s+K_{s,41}} \cdot \left(1 + 2 \cdot \frac{C_s}{C_s+K_{s,42}}\right) \cdot \frac{K_{sto,41}}{X_{sto}+K_{sto,41}}$	v_{41}
Storage release	$k_{42} \cdot X_{E,4} \cdot \frac{K_{s,42}}{C_s+K_{s,42}} \cdot \left(1 + 2 \cdot \frac{K_{s,41}}{C_s+K_{s,41}}\right) \cdot \frac{X_{sto}^2}{X_{sto}^2+K_{sto,42}^2} \cdot \frac{X_{ATP}^2}{X_{ATP}^2+K_{ATP,42}^2}$	v_{42}
Death rate	$v_{d,m}$	v_d

Table C3: Parameters of the $9 - pool$ model.

Param.	Value	St. Err.	Unit
$q_{E,11,max}$	$6.5 \cdot 10^{-2}$	$4.41 \cdot 10^{-3}$	$U/C\text{mol}_x/\text{h}$
μ_0	$5.5 \cdot 10^{-2}$	$1.03 \cdot 10^{-3}$	h^{-1}
k_{11}	0.10	$1.16 \cdot 10^{-3}$	h^{-1}
$k_{dE,11}$	$1.46 \cdot 10^{-2}$	$6.55 \cdot 10^{-3}$	h^{-1}
$k_{E,11}$	0.26	$1.54 \cdot 10^{-2}$	mol_s/U
$k_{s,11}$	$9.8 \cdot 10^{-6}$	$1.54 \cdot 10^{-2}$	mol/kg
$v_{12,max}$	0.18	$7.74 \cdot 10^{-2}$	$\text{mol}/C\text{mol}_x/\text{h}$
$K_{gly,12}$	31.38	4.91	$\mu\text{mol}/\text{g}_{dw}$
$K_{AA,12}$	870.23	69.6	$\mu\text{mol}/\text{g}_{dw}$
$K_{ATP,12}$	2.01	0.26	$\mu\text{mol}/\text{g}_{dw}$
$v_{13,max}$	0.32	$9.92 \cdot 10^{-2}$	$\text{mol}/C\text{mol}_x/\text{h}$
$K_{gly,13}$	38.54	9.25	$\mu\text{mol}/\text{g}_{dw}$
$K_{AA,13}$	757.81	128.83	$\mu\text{mol}/\text{g}_{dw}$
$K_{ATP,13}$	1.95	0.24	$\mu\text{mol}/\text{g}_{dw}$
$v_{21,max}$	0.35	$9.45 \cdot 10^{-2}$	$\text{mol}/C\text{mol}_x/\text{h}$
$K_{gly,21}$	25.64	5.58	$\mu\text{mol}/\text{g}_{dw}$
$K_{ATP,21}$	6.01	0.66	$\mu\text{mol}/\text{g}_{dw}$
$m_{ATP,22}$	$3.3 \cdot 10^{-2}$	$1.2 \cdot 10^{-2}$	$\text{mol}/C\text{mol}_x/\text{h}$
$k_{perm,31}$	$1.62 \cdot 10^{-2}$	$3.74 \cdot 10^{-3}$	m/h
a_{cell}	56.00	–	$\text{m}^2/C\text{mol}_x$
α_{32}	0	0	h^{-2}
β_{32}	$1.56 \cdot 10^{3}$	218.4	h^{-1}
$k_{dE,32}$	0.35	$4.90 \cdot 10^{-2}$	h^{-1}
β_{33}	$6.5 \cdot 10^{-4}$	–	$\text{mol}/C\text{mol}_x/\text{h}$
$k_{dE,33}$	$1.47 \cdot 10^{-2}$	$1.2 \cdot 10^{-3}$	h^{-1}
$K_{33,gly}$	30.76	1.58	$\mu\text{mol}/\text{g}_{dw}$
m_{33}	6.00	–	–

Table C4: Parameters of the $9 - pool$ model (continued)

Param.	Value	St. Err.	Unit
α_4	$8.01 \cdot 10^{-4}$	$1.68 \cdot 10^{-4}$	mol/Cmol$_x$/h^2
β_4	0.289	$1.49 \cdot 10^{-2}$	mol/Cmol$_x$/h
$k_{dE,4}$	0.29	$1.23 \cdot 10^{-2}$	h^{-1}
k_{41}	1.01	0.1	mol/mol
$K_{s,41}$	10^{-8}	$1.33 \cdot 10^{-8}$	mol/kg
$K_{sto,41}$	$4.25 \cdot 10^3$	$9.32 \cdot 10^2$	μmol/g$_{dw}$
k_{42}	3.99	0.48	mol/mol
$K_{s,42}$	10^{-4}	$1.37 \cdot 10^{-4}$	mol/kg
$K_{sto,42}$	$7.99 \cdot 10^3$	$1.07 \cdot 10^3$	μmol/g$_{dw}$
$K_{ATP,42}$	6.48	0.9	μmol/g$_{dw}$
pH_{int}	2.20	–	–
pH_{ext}	6.50	–	–
pK_{PAA}	4.31	–	–
$v_{d,m}$	$5 \cdot 10^{-3}$	–	h^{-1}
$M_{w,bio}$	28.05	–	g$_{dw}$/Cmol

Appendix E. Nomenclature

Abbreviations

**CFD**	Computational Fluid Dynamics
**CRD**	Computational Reaction Dynamics
**CM**	Compartment Model
**EL**	Euler-Lagrange
**PBM**	Population Balance Model
**RTD**	Residence Time Distribution
**SD**	Scale-Down
**STR**	Stirred Tank Reactor
**UDM**	User Defined Memory
**UDF**	User Defined Function

Roman	Units	Description
A	m^2	Area (general)
C	m	Impeller off-bottom clearance
ΔC	m	Impeller mutual clearance
C_s	mol/kg	Substrate concentration
$C_{s,c}$	mol/kg	Compartment subst. concentration vector
C_{ext}	mol/kg	Generic extracellular concentration
C_{int}	mol/g_{dw}	Generic intra-cellular concentration
C_x	g/kg	Biomass concentration (overall)
$C_{x,p}$	g/$parcel$	Biomass concentration (parcel)
D	m	Impeller diameter
$f_{c,face}$	kg/s	Flux through gridcell-face, convective
$f_{t,face}$	kg/s	Flux through gridcell-face, turbulent
F	kg/s	Feed rate (general)
1072 F_s	mol/s	Substrate feed rate (general)
H_l	m	Liquid filled tank height
K_s	mol/kg	Affinity constant for substrate
N_s	mol	Substrate amount
N_c	$-$	Total number compartments
N_{ax}	$-$	Axial compartments
N_r	$-$	Radial compartments
N_θ	$-$	Tangential compartments
N_p	$-$	Total number parcels
N_{liq}	$-$	Number of liquid phase species
N_{str}	RPM	Stirring speed
N_{pool}	$-$	Number of intra-cellular species
P_{jump}	$-$	Probability p leaves compartment i
$P_{dest,j}$	$-$	Probability p enters compartment j

**Roman**	**Units**	**Description**
q_p	$\text{mol}_p/C\text{mol}_x/\text{h}$	Specific production rate of product
q_s	$\text{mol}_s/C\text{g}_{dw}/\text{s}$	Specific uptake rate of substrate
$q_{s,max}$	$\text{mol}_s/C\text{g}_{dw}/\text{s}$	Max. Specific uptake rate of substrate
Q_{jump}	$-$	jump quantifier
r_p	$\mu\text{mol}/\text{g}_{dw}/\text{h}$	Reaction rate, parcel-based
$R_{s,c}$	mol/kg/s	Reaction rate of s, compartment-based
S	$-$	Stoichiometric matrix
1073 t	s	Time (general)
Δt_{max}	s	Maximum timestep size
Δt_{sample}	s	Sampling interval lifeline
T	m	Tank diameter
V	m^3	Tank volume
V_n	m^3	compartment volume
V_T	m^3	Total volume (general)
X_p	$\mu\text{mol}/\text{g}_{dw}$	Intra-cellular pool
Y_s	kg/kg	Mass fraction of s

**Greek**	**Units**	**Description**
ρ	kg/m^3	Density
μ	h^{-1}	Growth rate
Φ	m^3/s^{-1}	Flow matrix
ϕ_{ii}	m^3/s^{-1}	Flow out of compartment i, total
ϕ_{ij}	m^3/s^{-1}	Flow from compartment j to i, total
$\phi_{c,ij}$	kg/s	Flow from j to i, convective
$\phi_{t,ij}$	kg/s	Flow from j to i, turbulent
Φ	kg/s	Inter-compartment flow matrix, total
Φ_c	kg/s	Inter-compartment flow matrix, convective
Φ_t	kg/s	Inter-compartment flow matrix, turbulent
ψ	$-$	Uniform random number
τ_{95}	s	Mixing time
τ_{circ}	s	Circulation timescale
$\overline{\tau_{reg}}$	s	Regime residence time
τ_{rxn}	s	Uptake timescale of substrate
τ_{run}	s	Simulation runtime
τ_n	s	Residence time, compartment n
